# circ_0086296 induced atherosclerotic lesions via the IFIT1/STAT1 feedback loop by sponging miR-576-3p

**DOI:** 10.1186/s11658-022-00372-2

**Published:** 2022-09-23

**Authors:** Min Zhang, Yiqian Zhu, Jie Zhu, Yi Xie, Ruihao Wu, JiaYin Zhong, Zhaohui Qiu, Li Jiang

**Affiliations:** 1 Division of Cardiology, Tongren Hospital, Shanghai Jiao Tong University School of Medicine, Shanghai, China; 2 Department of Neurosurgery, Huashan Hospital, Fudan University, Shanghai, China; 3 Center for Translational Neurodegeneration and Regenerative Therapy, Tenth People’s Hospital of Tongji University, Shanghai, China

**Keywords:** Atherosclerosis, circ_0086296, miR-576-3p, IFIT1, STAT1, Exosomes, EIF4A3

## Abstract

Extensive inflammation of endothelial cells (ECs) facilitates atherosclerotic lesion formation. Circular RNA (circRNA) participates in atherosclerosis (AS)-related inflammation responses; however, whether and how circ_0086296 regulates atherosclerotic inflammation and lesions have not been investigated. Microarray analysis, quantitative real-time polymerase chain reaction, and fluorescence in situ hybridization assay were performed to detect the expression and location of hsa_circ_0086296 in human carotid artery plaques, aorta of atherosclerotic mice, and human umbilical vein endothelial cells (HUVECs). Sanger sequencing was used to verify the loop structure of circ_0086296. The relationship among circ_0086296, miR-576-3p, IFIT1, STAT1, and EIF4A3 was validated using bioinformatics, luciferase assay, RNA pull-down assay, and RNA immunoprecipitation. The atherosclerosis mouse model was used to evaluate the function of circ_0086296 in vivo. circ_0086296 expression was significantly upregulated in human carotid artery plaques, oxidized low-density lipoprotein (ox-LDL)-treated HUVECs, and the aorta of atherosclerotic mice. Functional analysis indicated that circ_0086296 promotes ECs injury in vitro and atherosclerosis progression in vivo. The mechanism analysis indicated that circ_0086296 sponged miR-576-3p to promote IFIT1–STAT1 expression. Moreover, STAT1 upregulated circ_0086296 expression, forming the circ_0086296/miR-576-3p/IFIT1/STAT1 feedback loop. Notably, inhibition of the circ_0086296/miR-576-3p/IFIT1 axis could block atherosclerotic lesion formation both in vivo and in vitro. Finally, circ_0086296 was overexpressed in exosomes of patients with atherosclerosis and exosomes of ox-LDL-treated ECs. Therefore, the circ_0086296/miR-576-3p/IFIT1/STAT1 feedback loop participates in atherosclerosis progression and contributes to the high circ_0086296 expression observed in the exosomes of serum of patients with atherosclerosis. This study sought to provide a deep understanding of the mechanisms underlying the aberrant EC phenotype in AS.

**Supplementary Information:**

The online version contains supplementary material available at 10.1186/s11658-022-00372-2.

## Introduction

Atherosclerosis (AS), described as a chronic and serious inflammatory response and injury in the arterial wall, is responsible for most serious cardiovascular diseases, including myocardial infarct and stroke [1–4]. Aberrant endothelial cells (ECs) loss and dysfunction is known as the initial step and one of the key pathological characteristics of AS [5–7]. Throughout the development of AS, ECs are subjected to numerous pathogenic pressures, especially oxidized low-density lipoprotein (ox-LDL), which facilitates endothelium breakdown, lipid accumulation, and atherosclerotic lesion formation [8]. Furthermore, injured ECs produce several types of cell factor, such as inflammatory cytokines, adhesion molecules, and matrix metalloproteinases, which can accelerate AS [9]. Consequently, understanding the molecular mechanisms underlying the atherosclerotic phenotype of ECs could highlight potential therapeutic targets for AS.

Circular RNAs (circRNAs) are generated through back-splicing of pre-mRNA transcripts [10, 11] and have a typical stable structure and long half-lives; thus, circRNAs might act as possible biomarkers in diseases [12, 13]. Increasing evidence has revealed that circRNAs mediate AS development via directly binding with protein or sponging miRNA to modify target gene levels [14–16]. circANRIL modulates the maturation of ribosomal RNA by binding with PES1, which blocks cellular proliferation and induces cell apoptosis in atherosclerotic plaques [17, 18]. In addition, abnormal circCHFR overexpression enhances the dysfunction of ox-LDL-treated vascular smooth muscle cells (VSMCs) by sponging miR-370, which eventually activates vascular remodeling [19]. Nevertheless, the mechanism by which circRNA biogenesis occurs and regulates the aberrant phenotype of ECs in AS remains unclear.

ISG56/interferon-induced protein with tetratricopeptide repeats 1 (IFIT1) is a member of the ISG56/IFIT1 family [20]. IFIT1 expression was increased in M1 polarized human primary macrophages; IFIT1 was also overexpressed in macrophages harvested from atherosclerotic mouse aorta [21]. Furthermore, IFIT1 participates in the atherosclerotic inflammatory response by generating interleukin (IL)-6, TNF-α, and NF-κB in vivo. Consequently, inhibition of IFIT1 may partly reduce the inflammatory response associated with AS development [22]. Thus, elucidating the underlying mechanism of IFIT1 expression may contribute to a deep understanding of the pathogenesis of AS.

Eukaryotic initiation factor 4A-III (EIF4A3), an RNA-binding protein (RBP), mediates exon splicing though binding with RNA and creating exon junction complexes (EJCs) [23]. circASAP1 expression stimulated by EIF4A3 elevates glioblastoma development via the ERK pathway [24], and E2F1- and EIF4A3-induced circSEPT9 expression accelerates triple-negative breast cancer progression [25]. Furthermore, circ_0084615 expression activated by EIF4A3 promotes colorectal cancer development by regulating miR-599 and onecut2 [26]. EIF4A3-induced circBNIP3 expression provokes hypoxia, causing H9C2 cell injury via targeting miR-27a-3p/BNIP3 [27]. Recently, a few reports revealed that hsa_circ_0030042 decreased EIF4A3, aggravating plaque instability in AS mice [28]. However, despite these findings, it remains unknown whether EIF4A3 can regulate circRNA biogenesis or whether EIF4A3 is involved in atherosclerotic lesion formation.

Interestingly, circRNAs may be present in exosomes and carried to the surrounding cells, thereby affecting the progression of diseases involving AS [29, 30]; exosomes have a diameter of < 150 nm and are secreted by numerous cell types, including ECs [31, 32]. However, knowledge of the detailed role of exosomes containing circRNAs in AS remains limited.

Therefore, we aimed to determine whether circ_0086296 is involved in AS inflammatory response and lesion development. This study sought to provide a deep understanding of the mechanisms underlying the aberrant EC phenotype in AS progression.

## Methods and materials

### Human carotid artery plaque tissues

Human carotid artery plaque tissues and control samples were collected from Shanghai Tongren Hospital. This study was approved by the ethics committee of Shanghai Tongren Hospital, and informed consent was obtained from patients prior to beginning the experiments (2019-091-01, 20 December 2019). The tissues were frozen in liquid nitrogen until use.

### Microarray assay

Total RNA from human carotid artery plaque samples and control tissues was extracted and subjected to microarray hybridization. Data analyses were performed using Agilent Human lncRNA Microarray 2018 (4*180k, DesignID: 085630) by OE Biotechnology Co., Ltd. (Shanghai, China) containing 19,247 probes for human mRNA, 15,561 probes for human lncRNAs, and 21,442 probes for human circRNA. The arrays were examined using Agilent Scanner G2505C (Agilent Technologies, Santa Clara, CA, USA).

### Sanger sequencing, actinomycin D, RNase R, and subcellular fraction analysis

The primers for the Sanger sequencing assay were produced by OE Biotechnology Co., Ltd. (Shanghai, China). For the actinomycin D assay, human umbilical vein endothelial cells (HUVECs) were treated with actinomycin D for 24 h. For the RNase R assay, total RNA was incubated with RNase R, and the stability of circRNA was assessed by quantitative real-time polymerase chain reaction (qRT-PCR). Cytoplasm and nucleus in HUVECs were separated using the PARIS Kit according to the manufacturer’s instructions, and the levels of circ_0086296 or UHRF2 were measured via qRT-PCR.

### Fluorescent in situ hybridization (FISH) analysis

To study the location of circ_0086296 in HUVECs and aortic tissues, a FISH kit was used (RiboBio, Guangzhou, China). First, all the sections were fixed in 4% paraformaldehyde and dehydrated with ethanol. Then, the sections were hybridized with Cy3-labeled circ_0086296 probes at 37 °C overnight and stained with DAPI. The sections were imaged using a confocal laser scanning microscope (Leica Microsystems, Wetzlar, Germany).

### Cell culture

HUVECs were obtained from AllCells (Shanghai, China) as previously reported [7, 33]. HUVECs were seeded in EC complete growth medium at 37 °C. Then, 100 μg/mL ox-LDL was added to the cells for 24 h, or continuous normal culture was conducted.

### Transient transfection of cells

circ_0086296 overexpression plasmid, circ_0086296 shRNA, miR-576-3p mimics/inhibitor, IFIT1 overexpression plasmid, IFIT1 shRNA, and the corresponding controls were purchased from Genomeditech (Shanghai, China). HUVECs were transfected with the circ_0086296 overexpression plasmid, shRNA of circ_0086296, miR-576-3p mimic, or miR-576-3p inhibitor using Lipofectamine 3000 (Invitrogen, Waltham, MA, USA) following the manufacturer’s instructions. The primers are listed in Additional file 2: Table S1.

### Cell Counting Kit 8 (CCK-8), Transwell migration, wound healing, and tube formation assays

Cell viability was determined using a CCK-8 kit (Dojindo, Shanghai, China), as previously described [7]. The migration ability of HUVECs was determined using a 12-well Boyden Transwell assay (8 μm, Corning, Glendale, AZ, USA), which was performed for 24 h. Then, cell motility was evaluated using a wound healing assay for 48 h. Finally, tube network formation analysis was conducted to reveal the angiogenic capability of HUVECs, as previously described [33]. Briefly, HUVECs were added into 96-well plates covered with 90 μL per well Matrigel (BD Biosciences, Franklin Lakes, NJ, USA) for 18 h, and the number of tube-like formations was calculated.

### qRT-PCR

Total RNA was harvested by TRIzol reagent (Invitrogen). Then, the total RNA was reverse transcribed by PrimeScript RT Master Mix (for mRNA and circRNA) or miScript II RT kit (for miRNA). qRT-PCR was performed using a GoTaq qPCR Master Mix (Promega, Madison, WI, USA) as previously described. The primers are presented in Additional file 2: Table S1.

### Western blotting

Proteins were extracted using RIPA buffer, separated in 10% SDS-PAGE, and transferred to PVDF membranes. The blots were blocked with 5% skim milk powder and treated with primary antibodies (Abcam, Shanghai, China) overnight. The membranes were then treated with HRP-linked secondary antibodies, and blots were visualized using an ECL kit (Millipore, Darmstadt, Germany).

### RNA pull-down assay and RNA immunoprecipitation

Biotin-labeled probe circ_0086296 flanking RNA sequences were obtained. Then, these flanking sequences were added into cell lysates and streptavidin magnetic beads, followed with analysis via western blotting. RIP assay was performed using the Magna RIP Kit (Millipore), as previously described.

### Luciferase activity assay

The miR-576-3p mimics and luciferase reporter plasmid were cotransfected into HEK293T cells by Lipofectamine 3000 reagent as previously described. The luciferase activity was assessed using a Dual Luciferase Assay Kit (Promega) following the manufacturer’s instructions.

### AS mouse model construction

SPF-grade ApoE^−/−^ mice (aged 24–28 days) were obtained from the Model Animal Research Center of Nanjing University and housed in specific-pathogen-free conditions. The study was approved by the animal ethics committee of Shanghai Tongren Hospital (2020-079, 2 March 2020). All procedures were performed in keeping with the standards set out in the Declaration of Helsinki and Laboratory Guidelines of Research in China and the National Institutes of Health Laboratory Animal Care and Use Guidelines. The mice were fed a high-fat diet (HFD) with 0.25% cholesterol and 15% fat for 16 weeks to develop the AS mouse model [7]. The control group was fed a normal diet (ND). Then, the six AS mice were injected with lentivirus expressing sh-NC, and another six AS mice were injected with lentivirus expressing sh-circ_0086296 via tail vein injection. After 42 days, all mice were sacrificed, and blood was collected. The aortas of the mice were immediately removed and stored with liquid nitrogen or fixed in 4% paraformaldehyde.

### Histopathology

Aorta tissue was fixed in 4% paraformaldehyde, and atherosclerotic plaques were visualized using Oil Red O staining, Masson trichrome staining, and hematoxylin–eosin (HE) staining, as previously described [6]. The area of atherosclerotic lesions was visualized under an optical microscope (Olympus, Tokyo, Japan).

### Immunohistochemistry (IHC) and immunofluorescence (IF) analysis

For IHC analysis, the samples were treated with primary antibodies at room temperature for 2 h, with secondary antibodies for 30 min, and then dyed with DAB and hematoxylin. For IF analysis, the paraffin sections were treated with primary antibodies overnight at 4 °C and fluorescent-conjugated secondary antibodies. The results were then visualized with a fluorescence microscope or confocal laser scanning microscope (Leica).

### Exosome isolation

Exosome were obtained from HUVEC culture medium or plasma, as previously described [34, 35]. Exosomes were detected by scanning electron microscopy and western blotting. Extracellular vesicle (EV) size distribution was analyzed using nanoparticle tracking analysis.

### Statistical analysis

All data were analyzed using SPSS 21.0 software (IBM Corp., Armonk, NY, USA). Measurement data were expressed as mean ± standard deviation. Two-group comparisons were performed using Student’s *t*-test. Data comparisons among multiple groups were performed using one-way analysis of variance (ANOVA) followed by Tukey’s post hoc test. A value of *p* < 0.05 was considered statistically significant.

## Results

### circ_0086296 is aberrantly expressed in human plaque tissues

**Fig. 1:**
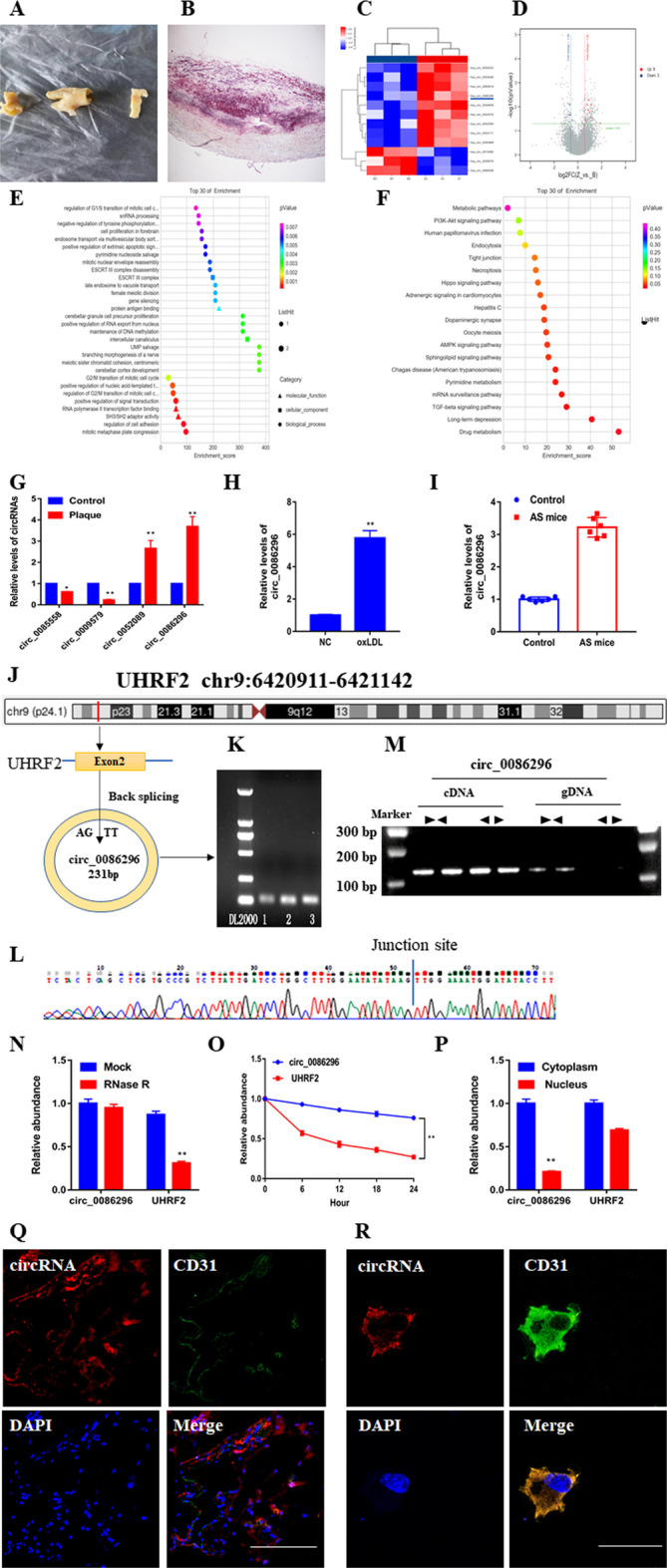
Validation and characterization of circ_0086296. **A** Morphology of human carotid atherosclerotic plaque tissues. **B** Human plaque tissue sections were stained with Oil Red O. **C** Cluster heatmap showing the abnormally expressed circRNAs from the microarray data. **D** Volcano map showing the abnormally expressed circRNAs. **E** Gene Ontology analysis of the abnormally expressed circRNAs. **F** Abnormally expressed circRNAs were identified by Kyoto Encyclopedia of Genes and Genomes pathway analysis. **G** The expression of abnormally expressed circRNAs in plaque samples. **H**, **I** Levels of circ_0086296 in HUVECs treated by oxidized low-density lipoprotein (ox-LDL) (100 μg/mL) for 24 h (**H**) and in the aorta of atherosclerotic mice (**I**) were determined via quantitative real-time polymerase chain reaction (qRT-PCR). **J** Graphic showing UHRF2 circularization to form circ_0086296. **K** The results of circ_0086296 PCR using agarose gel electrophoresis. **L** The back-splice junction sequences of circ_0086296 were found using Sanger sequencing. **M**–**P** circ_0086296 expression in HUVECs (**M**); in HUVECs after RNase R treatment (**N**); levels of circ_0086296 and UHRF2 after actinomycin D treatment (**O**); and levels of circ_0086296 and UHRF2 mRNA in the cytoplasm and nucleus of HUVECs (**P**) were determined via qRT-PCR. **Q**, **R** The localization of circ_0086296 in plaque tissues (scale bar, 50 μm) (**Q**) and localization of circ_0086296 in HUVECs (scale bar, 10 μm) (**R**) were verified via fluorescence in situ hybridization (FISH). Nuclei were stained with DAPI (blue), and circ_0086296 probes were labeled with Cy3 (red). *p < 0.05; **p < 0.01 versus the relative control group

Microarray analysis screened the circRNAs in the human plaque tissues and control samples (Fig. 1A, B). The results were presented as a heatmap after normalization (Fig. 1C). A volcano plot was used to display the highly or lowly expressed circRNA (*p* < 0.05; Fig. 1D). Gene Ontology (GO) analysis found that circRNAs are involved in several cellular processes, including regulation of cell adhesion (Fig. 1E). Pathway investigation revealed that these circRNAs were connected with AMPK signaling, TGF-β signaling pathway, etc. (Fig. 1F). Nine circRNAs had increased expression in human plaque tissues compared with the controls (Additional file 3: Table S2), whereas three circRNAs had decreased expression. Among these differentially expressed circRNAs, we chose four for further detection by qRT-PCR: circ_0086296, circ_0009579, circ_0085558, and circ_0052089 (< 2000 nt; Fig. 1G). Furthermore, our data showed that HUVECs have a higher circ_0086296 level than that of human aortic VSMCs or THP-1 cells (Additional file 1: Fig. S1A). qRT-PCR analysis indicated that treatment of HUVECs with ox-LDL could promote circ_0086296 expression (Fig. 1H). By using the CircBank and CircAtlas 2.0 database, we found that the circ_0086296 (mmu-circ_0000945) sequence is highly conserved in mice and humans (Additional file 1: Fig. S1B). The expression of mmu-circ_0000945 was upregulated in the heart, liver, and lungs of AS mice (Additional file 1: Fig. S1C). Furthermore, the level of circ_0086296 was upregulated in atherosclerotic mouse aortas compared with that in the control group (Fig. 1I).Our data analysis revealed that circ_0086296 was recognized as a transcript at the site of chromosome 9 (6,420,911–6,421,142) of the ubiquitin PHD RING finger 2 gene (*UHRF2*) (Fig. 1J, K). Sanger sequencing verified the head-to-tail splicing of circ_0086296 in HUVECs (Fig. 1L). In HUVECs, circ_0086296 could be amplified from only cDNA, whereas no product was found to be amplified from gDNA (Fig. 1M). We found that circ_0086296 was resistant to RNase R, indicating that circ_0086296 has high stability (Fig. 1N). At the same time, circ_0086296 had a notably long half-life (Fig. 1O). We also revealed that circ_0086296 was mostly expressed in the cytoplasm (Fig. 1P). Furthermore, FISH assay verified that circ_0086296 (red fluorescence) was colocalized with the endothelial-related marker CD31 in human coronary plaque tissues and artery tissues (Fig. 1Q and Additional file 1: Fig. S1D). In addition, we found that circ_0086296 was mostly located in cytoplasm of HUVECs (Fig. 1R). Collectively, the results showed that circ_0086296 expression is high in human plaque tissues and may have a key function in atherosclerotic plaque formation.

### circ_0086296 knockdown alleviates the atherosclerotic phenotype of HUVECs induced by ox-LDL in vitro

**Fig. 2:**
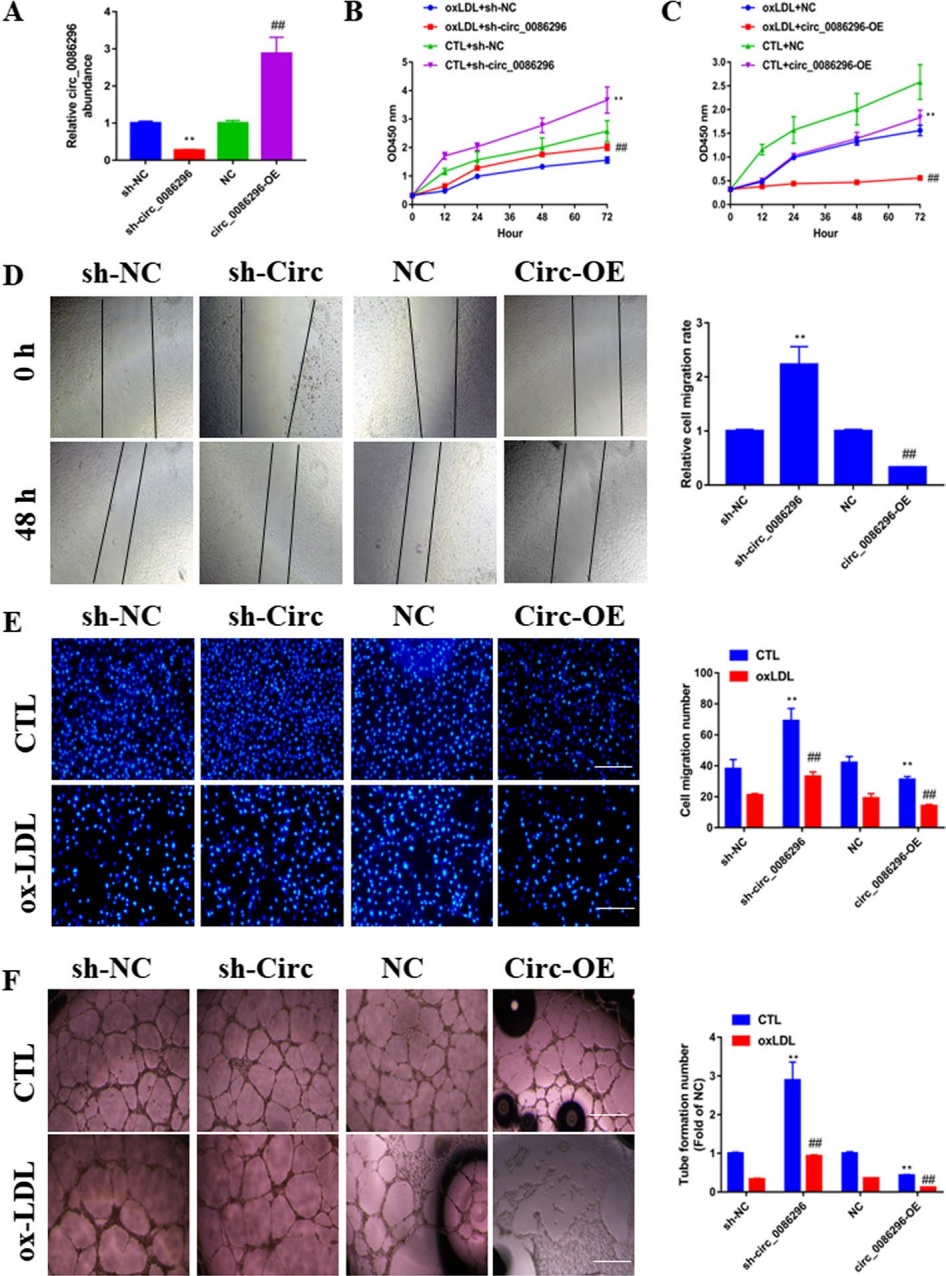
The effects of circ_0086296 on ox-LDL-induced atherosclerotic phenotype in HUVECs. **A** The effects of circ_0086296 overexpression or sh-circ_0086296 were measured via qRT-PCR. **B** The viability of HUVECs infected with sh-circ_0086296 vector. **p < 0.001 versus the sham + shNC group. ^##^*p* < 0.001 versus the oxLDL + shNC group. **C** The viability of HUVECs infected with circ_0086296 overexpression vector was determined via CCK-8 assay. **p < 0.001 versus the sham + shNC group. ^##^*p* < 0.001 versus the oxLDL + shNC group. **D** The migration of HUVECs infected by circ_0086296 overexpression vector or sh-circ_0086296 vectors was determined via wound healing assay. Scale bar, 100 μm. **p < 0.001 versus the shNC group. ^##^*p* < 0.001 versus the OE-NC group. **E** The migration of HUVECs transfected with circ_0086296 overexpression vector or sh-circ_0086296 vectors was determined via Transwell assay. Scale bar, 100 μm. **p < 0.001 versus the relative control group. ^##^*p* < 0.001 versus the untreated group. **F** The angiogenic ability of HUVECs infected with circ_0086296 overexpression vector or sh-circ_0086296 vectors was measured via Matrigel assay. **p < 0.001 versus the relative control group. ^##^*p* < 0.001 versus the untreated group

To identify the biological functions of circ_0086296 in the regulation of the HUVECs phenotype, we established stable circ_0086296 knockdown (sh-circ_0086296) and overexpression (circ_0086296-OE) cell lines, along with the respective controls (Fig. 2A). The qRT-PCR results showed that shRNA-circ specifically reduced the circ_0086296 expression, while having no effect on UHRF2 mRNA expression (Additional file 1: Fig. S1E, F). Thus, after knockdown of circ_0086296 with shRNA, there was no effect on its parent gene. Subsequently, CCK-8 assay revealed that circ_0086296 knockdown reversed the repressive effects of ox-LDL on HUVEC viability (Fig. 2B), and circ_0086296-OE increased these repressive effects (Fig. 2C). Next, Transwell and scratch assays illustrated that circ_0086296 knockdown alleviated ox-LDL-impaired HUVECs motility, whereas circ_0086296-OE cells had the opposite effect (Fig. 2D and E). Matrigel assay also found that circ_0086296 knockdown acted as an antagonist against ox-LDL-impaired HUVEC tube formation (Fig. 2F). Similarly, inflammatory factor expression was repressed or induced by circ_0086296 knockdown or overexpression in HUVECs, respectively (Additional file 1: Fig. S2A–C). Thus, our data highlight that circ_0086296 mediated the atherosclerotic phenotype of HUVECs induced by ox-LDL.

### EIF4A3 promotes circ_0086296 expression in HUVECs

**Fig. 3:**
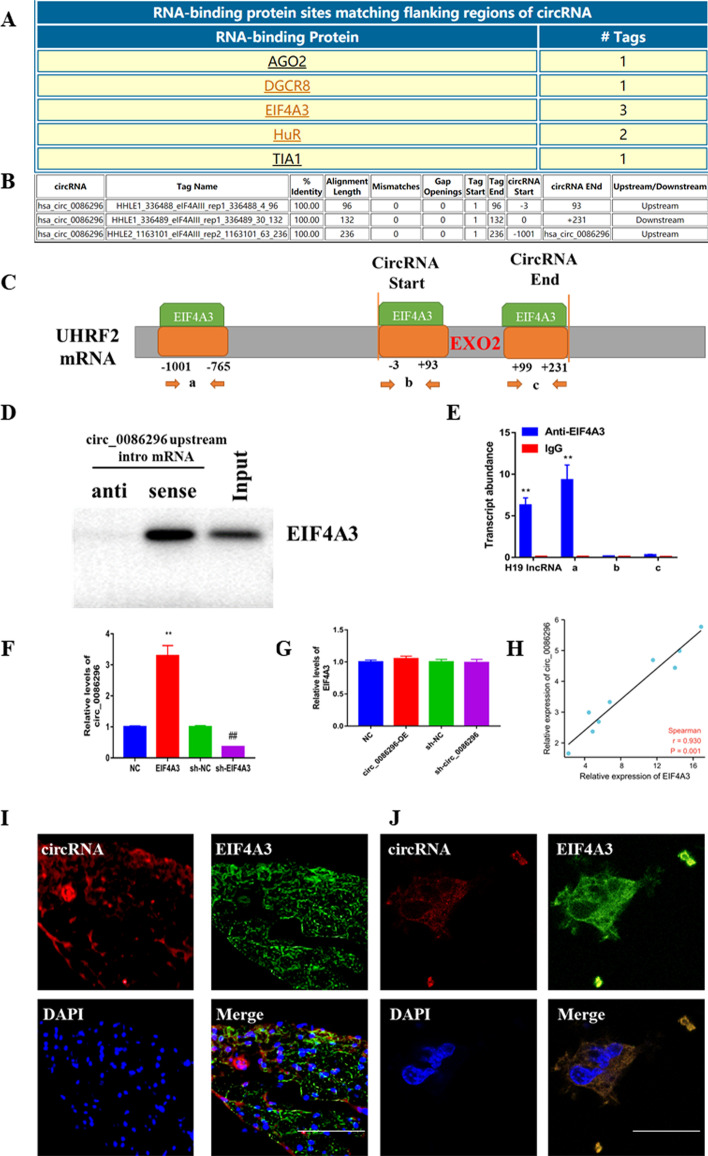
EIF4A3 induced circ_0086296 expression in HUVECs. **A**–**C** The potent area of EIF4A3 binding with the flanking sequences of the UHRF2 mRNA transcript was predicted. **D** The interaction between EIF4A3 and the circ_0086296 upstream region was detected. **E** RIP assay to demonstrate EIF4A3 binding with the putative sequences. **F** The levels of circ_0086296 in HUVECs transfected with sh-NC, sh-EIF4A3, pcDNA-NC, or pcDNA-EIF4A3, **p < 0.001 versus the OE-NC group. ^##^*p* < 0.001 versus the shNC group; and **G** the levels of EIF4A3 in HUVECs infected with circ_0086296 overexpression vector or sh-circ_0086296 vectors were determined via qRT-PCR. **H** The relationship between EIF4A3 and circ_0086296 was evaluated via Pearson’s correlation analysis (*n* = 9). **I**, **J** The colocalization of circ_0086296 and EIF4A3 in plaque tissues (**I**) (scale bar, 50 μm); and in HUVECs (**J**) was verified via fluorescence in situ hybridization (FISH) (scale bar, 10 μm). **p < 0.001, ^##^*p* < 0.001 versus the relative control group

RBP can directly bind with the flanking areas of circRNAs and mediate circRNA generation; thus, CircInteractome was used to evaluate the RBP of circ_0086296. There were three putative binding positions of EIF4A3 in the upstream and downstream areas of the UHRF2 mRNA transcript (circ_0086296 pre-mRNA) (Fig. 3A–C). Thus, EIF4A3 may bind with UHRF2 mRNA and EIF4A3 was chosen for further study. We then sought to verify whether EIF4A3 can combine with the flanking sequences of circ_0086296. The results demonstrated that EIF4A3 could combine with the upstream area of circ-UHRF2 pre-mRNA (Fig. 3D, E). To further explore the effects of EIF4A3 on circ_0086296 expression, EIF4A3 was overexpressed or knocked down in HUVECs (Additional file 1: Fig. S3A, B). We found that EIF4A3 overexpression facilitates circ_0086296 expression, whereas EIF4A3 knockdown inhibits circ_0086296 expression (Fig. 3F). EIF4A3 could increase ox-LDL-induced atherosclerotic phenotype in HUVECs (Additional file 1: Fig. S3C–E) and regulate miR-576-3p/IFIT1/STAT1 level (Additional file 1: Fig. S3F). Conversely, circ_0086296 overexpression or silencing did not change EIF4A3 expression (Fig. 3G). There was also a positive association between circ_0086296 and EIF4A3 expression in human plaque tissues (*n* = 9; Fig. 3H). Finally, we found that circ_0086296 was colocalized with EIF4A3 in human plaque tissues and HUVECs (Fig. 3I, J). Therefore, EIF4A3 might promote circ_0086296 expression by binding with its flanking sequences.

### circ_0086296 acts as an miR-576-3p sponge in HUVECs

**Fig. 4:**
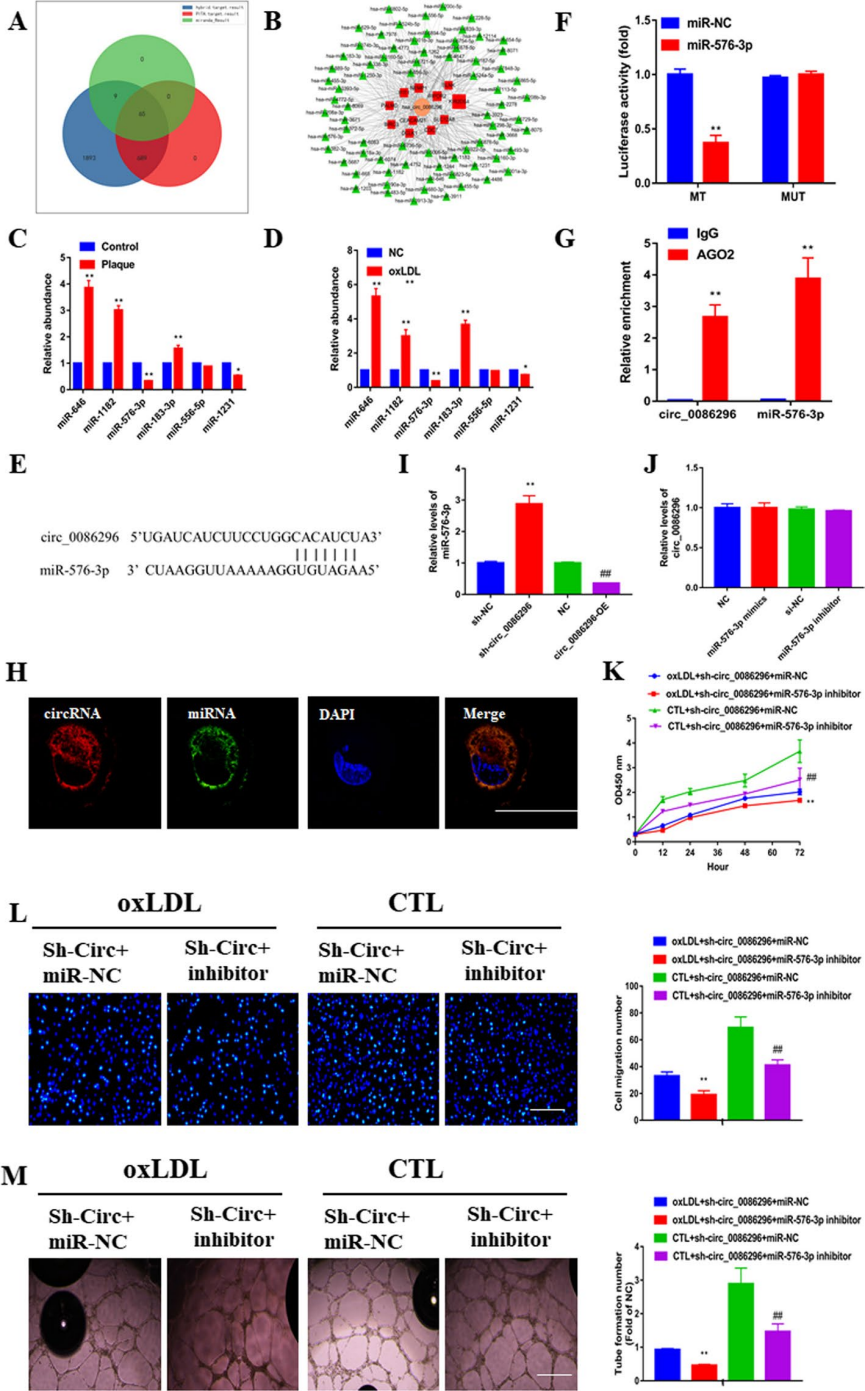
circ_0086296 serves as a sponge for miR-576-3p. **A** Graphic illustration displaying overlapping of the miRNAs target to circ_0086296 by RNAhybrid, PITA, and miRanda. **B** Schematic illustration showing the miRNA putative binding sequences with circ_0086296. **C** Relative levels of candidate miRNAs in plaque tissues were measured via qRT-PCR. *p < 0.05, **p < 0.001 versus the relative control group. **D** Relative expression of candidate miRNAs in HUVECs. *p < 0.05, **p < 0.001 versus the relative control group. **E** Schematic illustration presenting the putative binding area for miR-576-3p associated with circ_0086296. **F** The luciferase activity of circ_0086296 in HEK293T cells transfected with miR-576-3p mimics was verified via a luciferase reporter assay. **G** RNA immunoprecipitation (RIP) assay was performed using the anti-AGO2 antibody, and the enrichment of circ_0086296 and miR-576-3p was detected via qRT-PCR. **H** The colocalization of circ_0086296 and miR-576-3p in HUVECs was measured via fluorescence in situ hybridization (FISH) (scale bar, 10 μm). **I** Relative miR-576-3p expression levels in HUVECs infected with circ_0086296 overexpression vector or sh-circ_0086296 vectors were measured via qRT-PCR. **p < 0.001 versus the shNC group. ^##^*p* < 0.001 versus the OE-NC group. **J** Relative circ_0086296 expression in HUVECs infected with miR-576-3p mimics or inhibitors was measured. **K** The viability of HUVECs infected with sh-circ_0086296 and miR-576-3p inhibitor was determined via CCK-8 assay. **p < 0.001 versus the oxLDL + shcirc + miR-NC group. ^##^*p* < 0.001 versus the sham + shcirc + miR-NC group. **L** Detection of the migration potential of HUVECs infected with sh-circ_0086296 and miR-576-3p inhibitor. **p < 0.001 versus the oxLDL + shcirc + miR-NC group. ^##^*p* < 0.001 versus the sham + shcirc + miR-NC group. **M** Detection of the vasculogenic capability of HUVECs infected with sh-circ_0086296 and miR-576-3p inhibitor. **p < 0.001 versus the oxLDL + shcirc + miR-NC group. ^##^*p* < 0.001 versus the sham + shcirc + miR-NC group

As circ_0086296 is located in the cytoplasm of HUVECs, we examined whether it could act as a miRNA sponge. Bioinformatics analysis predicted the ceRNA networks of circ_0086296 (Fig. 4A, B; Additional file 4: Table S3, Additional file 5: Table S4 and Additional file 6: Table S5). A total of 65 miRNAs and 11 genes were selected through overlapping the datasets. Furthermore, miRNA expression was detected in human plaque tissues and ox-LDL-stimulated HUVECs (Fig. 4C, D). Notably, the results for miR-576-3p were of particular interest; the results exhibited that miR-576-3p inhibitor significantly suppressed the ability of angiogenesis in vitro, and the mimics reversed this effect (*p* < 0.05; Additional file 1: Fig. S4A–C); a schematic illustration demonstrated the putative binding sequences for miR-576-3p with circ_0086296 (Fig. 4E). Next, the dual luciferase reporter assay indicated that the circ_0086296 wild-type reporter (WT-circ_0086296) and miR-576-3p mimics reduced luciferase activity, whereas the circ_0086296 mutant-type reporter (MUT-circ_0086296) did not have this effect (Fig. 4F). RIP experiments also presented that that both miR-576-3p and circ_0086296 were enriched using the anti-AGO2 antibody (Fig. 4G). We then sought to determine the subcellular location of circ_0086296 and miR-576-3p in HUVECs. Our results indicated the colocalization of circ_0086296 with miR-576-3p in the cytoplasm of HUVECs (Fig. 4H). Furthermore, we found that circ_0086296 overexpression or inhibition could downregulate or upregulate miR-576-3p expression, respectively (Fig. 4I); meanwhile, miR-576-3p did not have an impact on circ_0086296 expression in ox-LDL-treated HUVECs (Fig. 4J). Thus, we verified that circ_0086296 functions as a molecular sponge of miR-576-3p.

### miR-576-3p reverses the atherosclerotic roles of circ_0086296 in HUVECs

To determine whether circ_0086296 promoted EC atherosclerotic injury via miR-576-3p, HUVECs were cotransfected with sh-circ_0086296 and miR-576-3p inhibitor, and functional assays were conducted. We found that miR-576-3p inhibitor could mitigate the promotion of cell viability by sh-circ_0086296 in ox-LDL-treated HUVECs (Fig. 4K). Similarly, miR-576-3p inhibition reversed the migration of HUVECs induction caused by circ_0086296 knockdown (Fig. 4L). Likewise, cotransfection of HUVECs with sh-circ_0086296 and miR-576-3p inhibitor significantly reduced capillary network formation (Fig. 4M). Moreover, the miR-576-3p inhibitor reversed the inhibition of inflammatory factor expression caused by circ_0086296 knockdown in oxLDL-treated HUVECs (Additional file 1: Fig. S5A–C). Taken together, these data show that circ_0086296 promotes the atherosclerotic phenotype of HUVECs via sponging miR-576-3p.

### IFIT1 is a direct target of miR-576-3p and functions as a promoter in AS

**Fig. 5:**
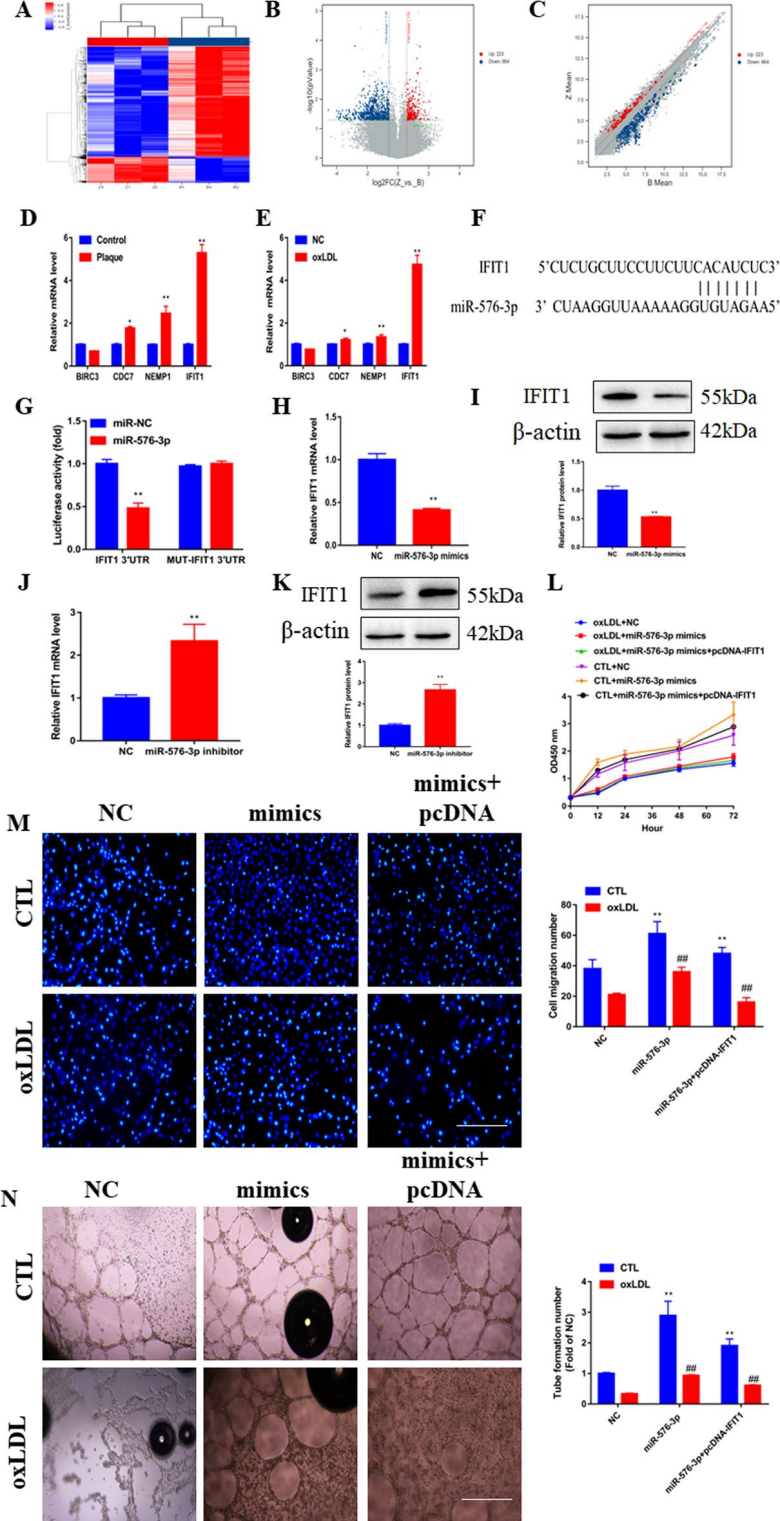
miR-576-3p mediates ox-LDL-induced HUVECs damage phenotype by targeting IFIT1. **A** Cluster heatmap exhibiting the abnormally expressed mRNAs from the microarray data. **B** Volcano map showing the abnormally expressed mRNAs. **C** Scatter plot presenting the abnormally expressed mRNAs from the microarray data. **D** Relative mRNA expression of IFIT1 was assessed in plaque tissues. *p < 0.05, **p < 0.001 versus the relative control group. **E** Relative mRNA expression of IFIT1 was assessed in ox-LDL-treated HUVECs. *p < 0.05, **p < 0.001 versus the relative control group. **F** Schematic illustration of the putative binding area for miR-576-3p associated with IFIT1. **G** Luciferase activity was revealed in cells coinfected with IFIT1-WT or IFIT1-MUT and miR-576-3p mimics and normal control (NC), respectively. **H** Relative IFIT1 mRNA levels were assessed in HUVECs via infection with miR-576-3p mimics and NC. **I** Relative IFIT1 protein expression was assessed in HUVECs via transfection with miR-576-3p mimics and NC. **p < 0.001 versus the NC group. **J** Relative IFIT1 mRNA levels were assessed in HUVECs via infection with miR-576-3p inhibitor and NC. **K** Relative IFIT1 protein expression was assessed in HUVECs via transfection with miR-576-3p inhibitor and NC. **p < 0.001 versus the NC group. **L** Viability of HUVECs infected with miR-576-3p mimics and overexpressed IFIT1 vector via CCK-8 assay. **M** Detection of migration potential of HUVECs infected with miR-576-3p mimics and overexpressed IFIT1 vector. **N** Detection of the vasculogenic capacity of HUVECs infected with miR-576-3p mimics and overexpressed IFIT1 vector. **p < 0.001 versus sham group, ^##^*p* < 0.001 versus the relative control group

Consistent with the ceRNA theory, circ_0086296 is positively associated with target gene levels, whereas it is negatively associated with miR-576-3p. We predicted the possible miR-576-3p target genes (Fig. 4A, B), and the genes from microarray analysis were also investigated (Fig. 5A–C). To further explore the key biological pathway associated with atherosclerotic plaque formation, the differentially expressed genes between the human plaque tissues and control samples were analyzed using KEGG analysis and gene set enrichment analysis (GSEA). The results of these analyses revealed that the pathways of “NF-κB signaling,” “hypertrophic cardiomyopathy,” “cytokine–cytokine receptor interaction,” and “dilated cardiomyopathy” were enriched (Additional file 1: Fig. S6). Four genes (*BIRC3*, *CDC7*, *NEMP1*, and *IFIT1*) were selected from the possible miR-576-3p target genes (Fig. 4B), and the results of qRT-PCR showed that IFIT1 was significantly upregulated in human plaque tissues and ox-LDL-treated HUVECs (Fig. 5D, E). Thus, IFIT1 was selected for further analyses. Our results exhibited that overexpressed IFIT1 significantly suppressed the ability of angiogenesis in vitro and sh-IFIT1 reversed this effect (Additional file 1: Fig. S7A–E). The schematic illustration indicated the putative binding sequences for miR-576-3p with IFIT1 (Fig. 5F). To determine whether IFIT1 was a potent target of miR-576-3p, the IFIT1 3′ untranslated region (UTR) luciferase reporter (IFIT1-WT) and the mutation reporter (IFIT1-MUT) were used. We found that IFIT1-WT and the miR-576-3p mimics downregulated luciferase activity, whereas IFIT1-MUT did not have this effect (Fig. 5G).

Subsequently, we detected how miR-576-3p modulated IFIT1 expression in ox-LDL-treated HUVECs. Unsurprisingly, the IFIT1 level was reduced by miR-576-3p mimics and elevated by anti-miR-576-3p (Fig. 5H–K). To examine how miR-576-3p affects IFIT1 and modulates the ECs phenotype, we overexpressed IFIT1 in ox-LDL treated HUVECs transfected with miR-576-3p mimics. We found that IFIT1 overexpression blocks the inhibitory roles of miR-576-3p in the HUVECs with the AS phenotype (Fig. 5L–N). Taken together, our results demonstrated that IFIT1 is a direct target of miR-576-3p and induces the phenotype of HUVECs damage caused by ox-LDL.

### circ_0086296 facilitates the atherosclerotic EC phenotype by targeting IFIT1 via miR-576-3p

**Fig. 6:**
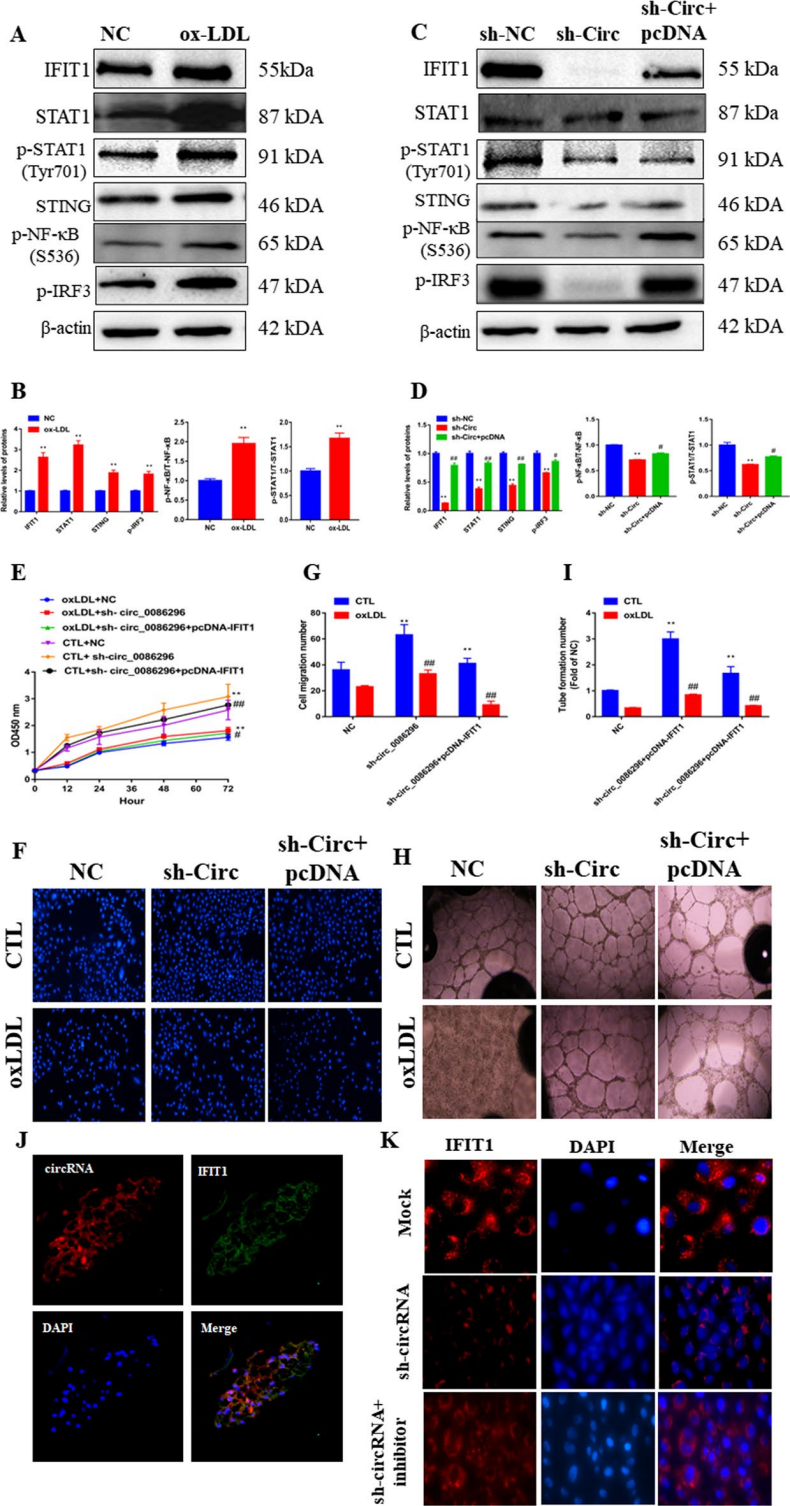
circ_0086296 acts as a miR-576-3p sponge to regulate IFIT1 levels. **A**, **B** Relative protein expression was measured in HUVECs treated with ox-LDL. **p < 0.001 versus sham group. **C**, **D** Relative protein expression was measured in HUVECs infected with sh-circ_0086296 vector and overexpressed IFIT1 vector. **p < 0.001 versus shNC group, ^#^*p* < 0.05, ^##^*p* < 0.001 versus the shcirc group. **E** Viability of HUVECs infected with sh-circ_0086296 and overexpressed IFIT1 vector via CCK-8 assay. **F**, **G** Detection of migration potential of HUVECs infected with sh-circ_0086296 and overexpressed IFIT1 vector. **p < 0.001 versus sham group, ^##^*p* < 0.001 versus the relative control group. **H**, **I** Detection of the vasculogenic capacity of HUVECs infected with sh-circ_0086296 and overexpressed IFIT1 vector. **p < 0.001 versus sham group, ^##^*p* < 0.001 versus the relative control group. **J** The colocalization of circ_0086296 and IFIT1 was measured in plaque tissues via fluorescence in situ hybridization (FISH). **K** IF staining displayed IFIT1 expression in HUVECs infected with sh-circ_0086296, and miR-576-3p inhibitor

To explore how circ_0086296 promoted the atherosclerotic lesions of HUVECs by regulating IFIT1, the IFIT1 overexpression plasmid and sh-circ_0086296 were transfected into HUVECs and the phenotypic changes of HUVECs were measured. Ox-LDL markedly induced IFIT1, STAT1, and STING expression and increased STAT1, NF-κB, and IRF3 phosphorylation in HUVECs (Fig. 6A, B). The levels of the above proteins were estimated in ox-LDL-treated HUVECs by loss of function of circ_0086296. The data showed that downregulation of circ_0086296 caused lower IFIT1 expression (Fig. 6C, D). Furthermore, the IFIT1 overexpression plasmid could reverse this effect (Fig. 6C, D). Downregulation of circ_0086296 was accompanied by the suppression of STAT1 and STING expression and attenuation of STAT1, NF-κB, and IRF3 phosphorylation in cells, and IFIT1 overexpression could reverse these effects (Fig. 6C, D).

Next, IFIT1 overexpression could mitigate the promotion of ox-LDL-treated HUVEC viability (Fig. 6E) and migration induced by sh-circ_0086296 (Fig. 6F, G). Furthermore, IFIT1 overexpression could negate the promotion roles of sh-circ_0086296 in capillary network formation (Fig. 6H, I). Compared with that in the sh-circ_0086296 group, inflammatory factor expression in ox-LDL-treated HUVECs was increased in the groups that were cotransfected with sh-circ_0086296 and overexpressing the IFIT1 plasmid group (Additional file 1: Fig. S8A–C).

Additionally, circ_0086296 (red fluorescence) and IFIT1 (green fluorescence) were colocalized in human coronary plaque tissues (Fig. 6J). Furthermore, IF staining displayed that sh-circ_0086296 significantly decreased IFIT1 expression, and this effect could be abolished by miR-576-3p inhibitor (Fig. 6K). These findings implied that circ_0086296 promotes the atherosclerotic lesion phenotype of HUVECs by upregulating IFIT1 expression via miR-576-3p.

### circ_0086296/miR-576-3p/IFIT1/STAT1 feedback loop regulates the atherosclerotic EC phenotype

**Fig. 7:**
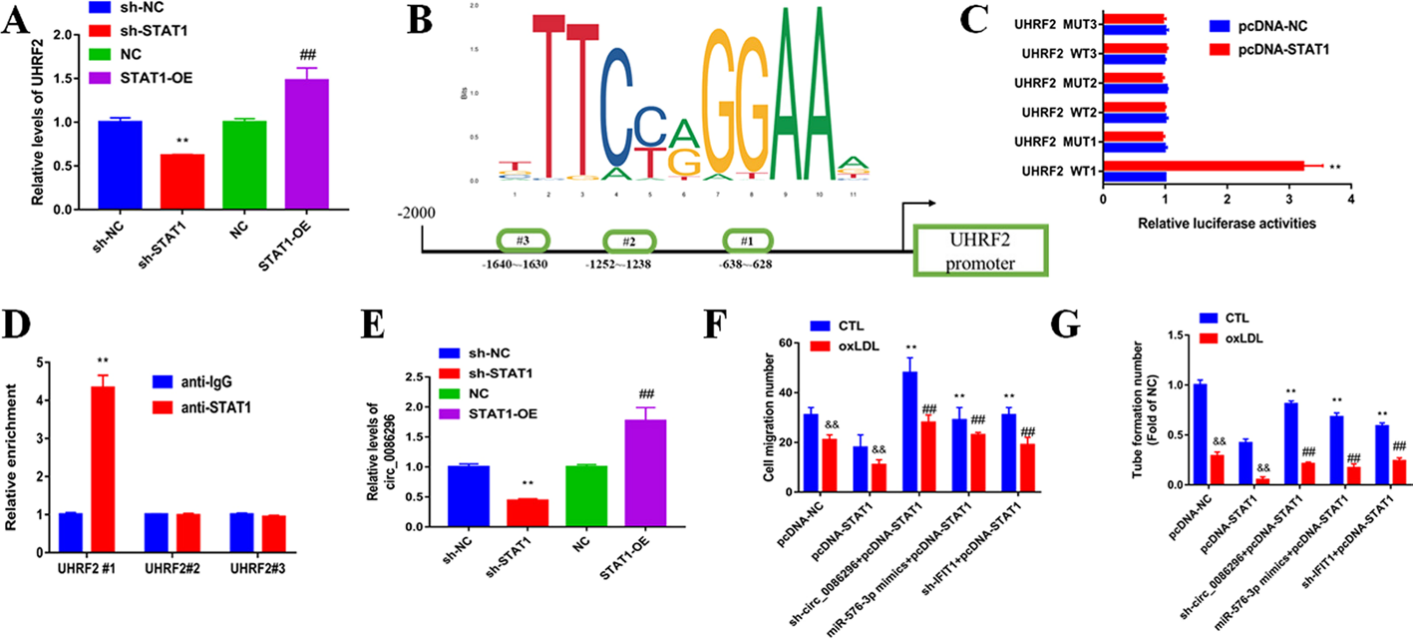
circ_0086296 is transcriptionally regulated by STAT1 in HUVECs. **A** UHRF2 level in HUVECs infected with sh-STAT1 vector and overexpressed STAT1 vector was detected by qRT-PCR. **p < 0.001 versus the shNC group. ^##^*p* < 0.001 versus the OE-NC group. **B** Schematic diagram exhibiting the binding sequences in the promoter area of circ_0086296 for STAT1. **C** The luciferase activities were measured using luciferase reporter assay in HUVECs infected with the UHRF2 vector (wild type and mutant) and overexpressed STAT1 vector. **D** The enrichment of potential binding sites were detected using the STAT1 antibody by ChIP–PCR. **E** Relative circ_0086296 expression was measured in HUVECs infected with sh-STAT1 vector and overexpressed STAT1 vector. **p < 0.001 versus the shNC group. ^##^*p* < 0.001 versus the OE-NC group. **F** Detection of migration potential of HUVECs infected with overexpressed STAT1 vector and sh-circ_0086296, miR-576-3p mimics, or sh-IFIT1. ^&&^*p* < 0.001 versus the sham group, **p < 0.001 versus the pcDNA group, ^##^*p* < 0.001 versus the relative control group. **G** Detection of the vasculogenic capacity of HUVECs infected with overexpressed STAT1 vector and sh-circ_0086296, miR-576-3p mimics, or sh-IFIT1. ^&&^*p* < 0.001 versus the sham group, **p < 0.001 versus the pcDNA group, ^##^*p* < 0.001 versus the relative control group

STAT1 can modulate the expression of target genes at the transcriptional level by combining with the promoter area. Our data showed that UHRF2 expression was increased in HUVECs by STAT1 overexpression, while sh-STAT1 could reduce UHRF2 expression (Fig. 7A). We found the potent STAT1-binding sites on the UHRF2 promoter using JASPAR, and three binding sequences were chosen for further research (Fig. 7B). Subsequently, luciferase reporter results revealed that STAT1 could induce WT-UHRF2 promoter luciferase activity but not MUT-UHRF2 promoter activity (Fig. 7C). Moreover, ChIP-qPCR assay results indicated that STAT1 could combine with the UHRF2 promoter and upregulate the transcriptional activity (Fig. 7D). Nevertheless, whether STAT1 can regulate circ_0086296 expression remains unclear, and our data showed that STAT1 overexpression enhanced circ_0086296 expression, while the repression of STAT1 blocked circ_0086296 expression (Fig. 7E). In brief, our data revealed that circ_0086296 could be transcriptionally modulated by STAT1 in HUVECs.

To identify the role of the circ_0086296/miR-576-3p/IFIT1/STAT1 feedback loop for the atherosclerotic EC phenotype, HUVEC function experiments were performed. STAT1 overexpression induced the atherosclerotic EC phenotype (Fig. 7F, G). Next, cotransfection of sh-circ_0086296, miR-576-3p mimics, or sh-IFIT1 could restore the atherosclerotic EC characteristics (Fig. 7F, G). Thus, these findings identify the role of the circ_0086296/miR-576-3p/IFIT1/STAT1 feedback loop for the atherosclerotic EC phenotype.

### circ_0086296 silencing inhibits atherosclerotic lesions in vivo

**Fig. 8:**
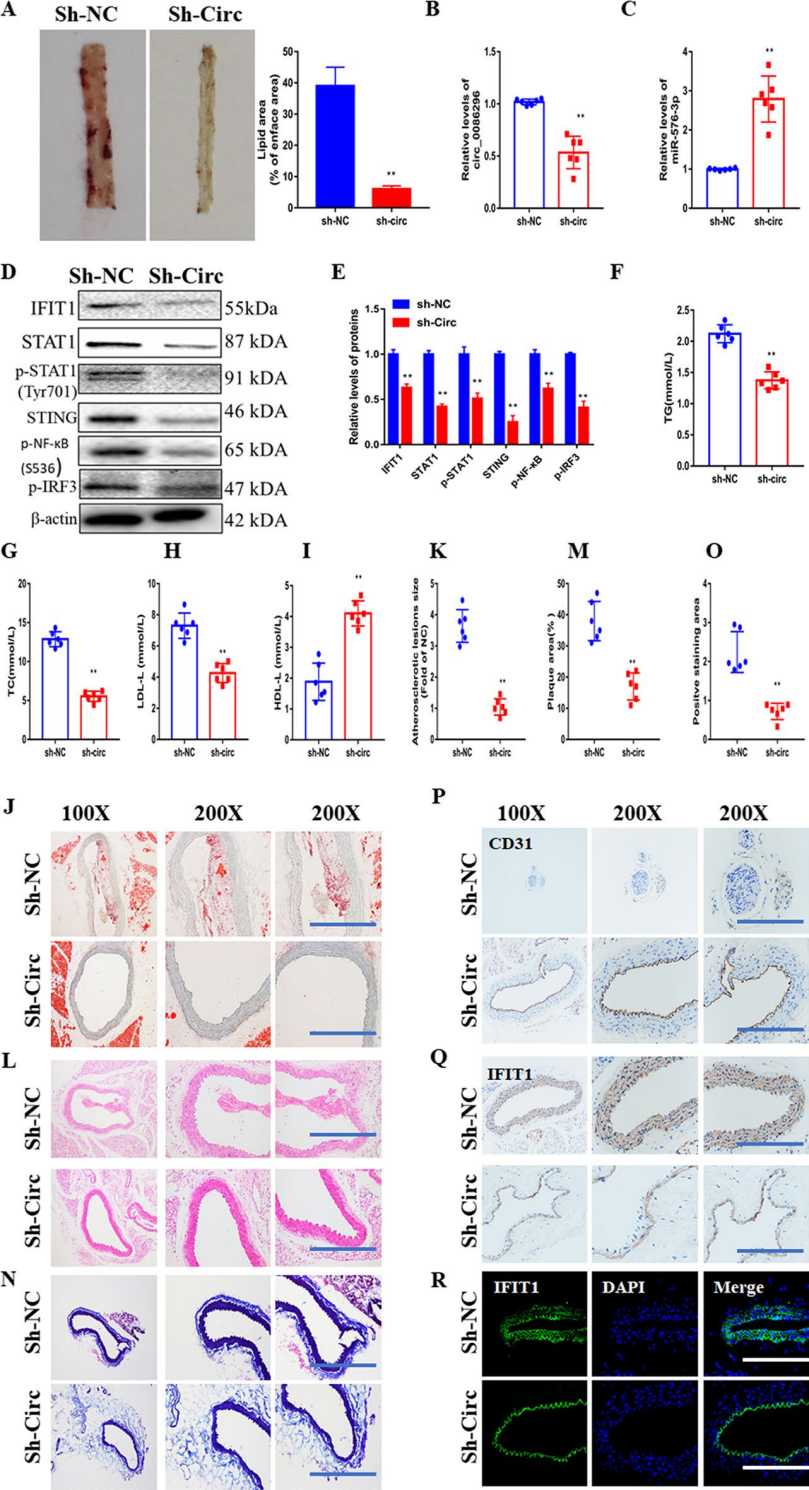
circ_0086296 causes the atherosclerotic phenotype in vivo. **A** Atherosclerotic mice were injected with sh-circ_008629, and the mice aortas were visualized with en face Oil Red O staining. **B** Relative circ_0086296 levels were detected in atherosclerotic mice injected with sh-circ_008629 or sh-NC. **C** Relative miR-576-3p levels were detected in atherosclerotic mice injected with sh-circ_008629 or sh-NC. **D** Relative IFIT1, STAT1, STING, p-STAT1, p-NF-κB, and pIRF3 levels were detected in atherosclerotic mice injected with sh-circ_008629 or sh-NC. **E** Quantification of **D**. **F**–**I** The levels of total cholesterol, triglyceride, and LDL-C were decreased and HDL-C was increased in atherosclerotic mice injected with sh-circ_008629. **J**, **K** Illustrative en face Oil Red O staining in atherosclerotic mice injected with sh-circ_008629 or sh-NC aorta. **L**, **M** Illustrative hematoxylin and eosin staining in atherosclerotic mice injected with sh-circ_008629 or sh-NC aorta. **N**, **O** Illustrative Masson staining in atherosclerotic mice injected with sh-circ_008629 or sh-NC aorta. **P** Immunohistochemical staining of CD31 in atherosclerotic mice injected with sh-circ_008629 or sh-NC aorta. **Q** Immunohistochemical staining of IFIT1 in atherosclerotic mice injected with sh-circ_008629 or sh-NC aorta. **R** Immunofluorescence staining of IFIT1 in atherosclerotic mice injected with sh-circ_008629 or sh-NC aorta. **p < 0.001 versus shNC group

The levels of circ_00862961 and IFIT1 were upregulated in the aortas of ApoE^−/−^ mice fed with HFD compared with those in the controls, whereas miR-576-3p expression was downregulated in the aorta of ApoE^−/−^ mice fed with HFD (AS mice; Additional file 1: Fig. S9A, B). To further elucidate the roles of circ_0086296 in promoting atherosclerotic lesions in vivo, the atherosclerotic mice were injected with lentiviruses expressing sh-NC or sh-circ_0086296. We found that sh-circ_0086296 reduced the lipid area of the aorta tissues of atherosclerotic mice (Fig. 8A). Infection with sh-circ_0086296 inhibited circ_0086296 expression (Fig. 8B), increased miR-576-3p expression (Fig. 8C), and decreased IFIT1 levels (Fig. 8D, E) in aorta tissues of atherosclerotic mice. To explore the molecular mechanism behind the repressing role of sh-circ_0086296 in atherogenesis in vivo, the STAT1–STING signaling in the aorta of atherosclerotic mice was measured. Both in vivo and in vitro data revealed that downregulation of circ_0086296 mediates the suppression of STAT1 and STING expression as well as the repression of STAT1, NF-κB, and IRF3 phosphorylation (Fig. 8D, E). More importantly, total cholesterol, triglyceride, and LDL-C were significantly decreased, and HDL-C was significantly increased in the plasma of AS mice after downregulation of circ_0086296 (Fig. 8F–I).

Additionally, Oil Red O, HE, and Masson staining of the aorta paraffin sections displayed that AS mice had atherosclerotic plaque formation and intima lesions (Fig. 8J–O). Interestingly, the areas of plaque and intima damage were partially inhibited in AS mice infected with lentivirus expressing sh-circ_0086296 (Fig. 8J–O). Furthermore, IHC showed that sh-circ_0086296 increased CD31 expression (Fig. 8P) and decreased IFIT1 expression (Fig. 8Q) compared with the sh-NC group. The IF results of IFIT1 were in line with those of IHC (Fig. 8R).

Meanwhile, ELISA showed that the levels of inflammatory factors significantly decreased after infection with sh-circ_0086296 in AS mice compared with the sh-NC group (Additional file 1: Fig. S9C–E). These data collectively suggested that circ_0086296 deficiency could limit atherosclerotic lesion development in vivo.

### circ_0086296 expression occurs in plasma EVs from patients with AS

**Fig. 9:**
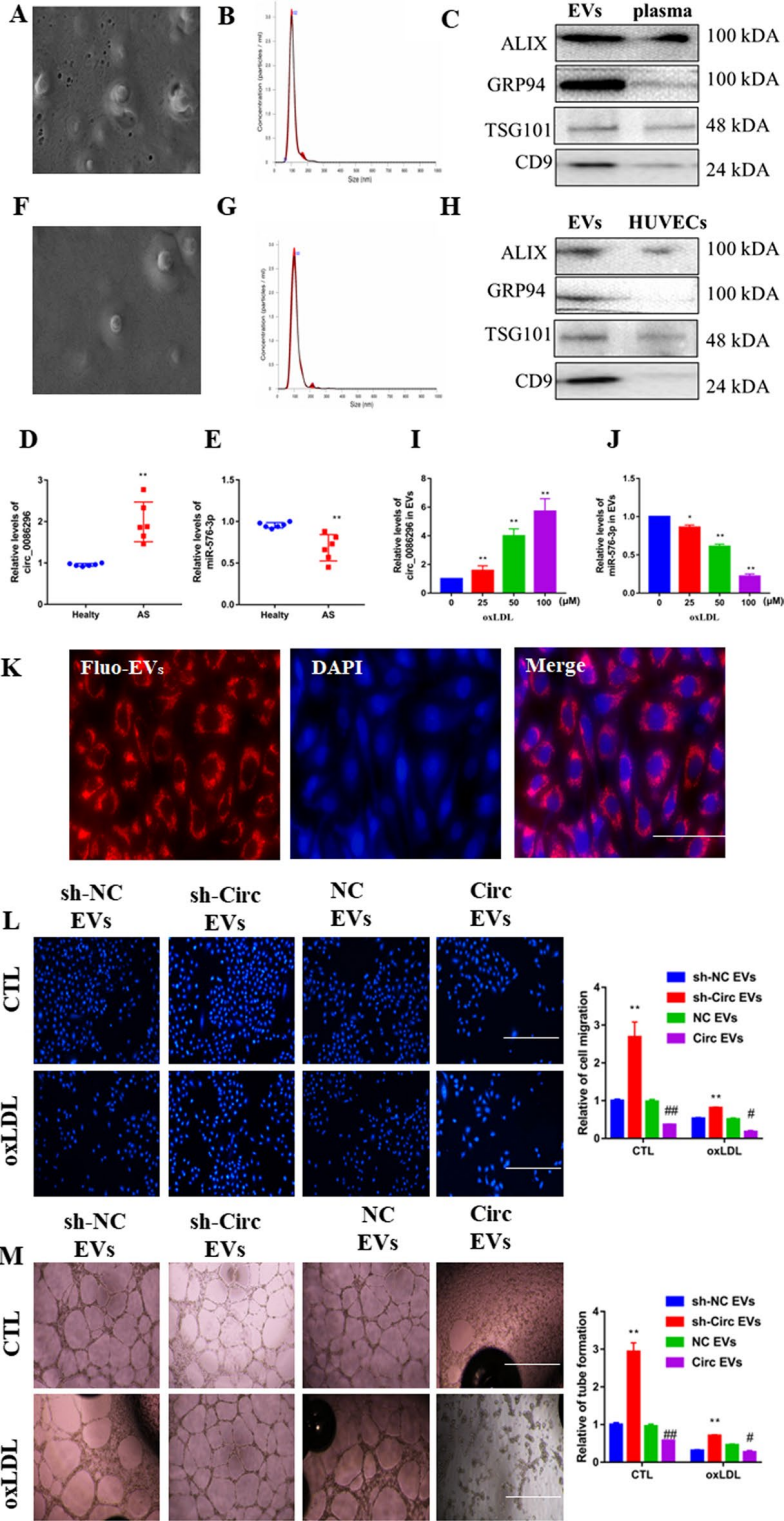
circ_0086296 induced the atherosclerotic lesion phenotype of HUVECs via exosomes. **A** Exosomes originating from plasma from patients with atherosclerosis were identified via scanning electron microscopy (SEM). **B** The size range of isolated extracellular vesicles (EVs) was calculated via Nanosight. **C** EV marker expression was revealed by western blot. **D**, **E** circ_0086296 (**D**) and miR-576-3p (**E**) expression in EVs originating from plasma from patients with atherosclerosis was detected via qRT-PCR. **p < 0.001 versus healthy group. **F** Exosomes originating from ox-LDL-treated HUVECs were identified via SEM. **G** The size range of isolated EVs was calculated via Nanosight. **H** The levels of EV markers were revealed by western blot. **I** circ_0086296 expression in EVs originating from ox-LDL-treated HUVECs was measured via qRT-PCR. **p < 0.001 versus sham group. **J** miR-576-3p expression in EVs originating from ox-LDL-treated HUVECs was detected via qRT-PCR. **K** Exosomes derived from the fluorescently labeled cells were taken up by HUVECs. **L** Migration potential of HUVECs treated with circ_0086296-OE and sh-circ_0086296 cell-derived exosomes was detected via Transwell assay. Scale bar, 100 μm. **p < 0.001 versus the shNC EV group, ^#^*p* < 0.05, ^##^*p* < 0.001 versus the OE-NC EV group. **M** Detection of the vasculogenic ability of HUVECs treated with circ_0086296-OE and sh-circ_0086296 cell-derived exosomes. *p < 0.05, **p < 0.001 versus the shNC EVs group, ^#^*p* < 0.05, ^##^*p* < 0.001 versus OE-NC EVs group

As EVs package circRNAs involved in AS, we purified exosomes from the plasma of six patients with AS and six control subjects (Fig. 9A). The sizes of the EVs from these samples were revealed with a size peak of 102 nm using Nanosight (Fig. 9B). The diameter of most of the microvesicles was < 150 nm (Fig. 9B). We also characterized EV markers, including ALIX, GRP94, TSG101, and CD9 (Fig. 9C). Higher levels of circ_0086296 were found in EVs of patients with AS (Fig. 9D), whereas miR-576-3p levels were reduced in the EVs of patients with AS compared with those of control subjects (Fig. 9E).

To study whether HUVECs secreted EVs containing circ_0086296, EVs derived from HUVEC medium were harvested (Fig. 9F). EVs were approximately 100 nm in size (Fig. 9G). EV markers, including ALIX, GRP94, TSG101, and CD9, were also measured (Fig. 9H). Similar to the in vivo results, we found that ox-LDL-treated HUVECs had increased circ_0086296 levels in EVs compared with the controls (Fig. 9I), whereas they had lower miR-576-3p expression (Fig. 9J). To determine whether EVs were taken up by the receptor cells, nucleic acids in EVs were dyed with red fluorescence. These EVs were cocultured with HUVECs, and fluorescently labeled EVs were observed in HUVECs (Fig. 9K).

We further investigated how the packaging of circ_0086296 by EVs regulates the atherosclerotic lesion phenotype of HUVECs. We found that EVs containing sh-circ_0086296 could partially recover the atherosclerotic lesion phenotype of HUVECs (Fig. 9L, M), whereas EVs containing circ_0086296-OE induced the atherosclerotic lesion phenotype (Fig. 9L, M).

circ_0086296 and miR-576-3p expression in cells after coculture with sh-circ_0086296 cell-derived EVs was detected using qPCR. The results showed that the expression of circ_0086296 decreased in cells that were cocultured with sh-circ_0086296 cell-derived EVs (Additional file 1: Fig. S10A). miR-576-3p expression increased in HUVECs after coculture with sh-circ_0086296 cell-derived EVs (Additional file 1: Fig. S10B). Overall, our results implied that EVs containing circ_0086296 accelerate the formation of the AS lesion phenotype of HUVECs.

## Discussion

Increasing evidence has implied that the dysregulation of circRNAs is related to atherosclerotic lesion formation and could serve as potential drug targets. This study reports three new findings: (a) EIF4A3-mediated circ_0086296 induces the development of atherosclerotic lesions in vitro and in vivo; (b) the circ_0086296/miR-576-3p/IFIT1/STAT1 feedback loop is involved in atherosclerotic lesion progression;and (c) the packaging of circ_0086296 by EVs accelerates the atherosclerotic lesion phenotype of ECs. Taken together, our results found that circ_0086296 may mediate the development of atherosclerotic lesions.

Several recent studies have revealed that circRNAs act as crucial regulators in AS [36], myocardial infarction [37–39], and heart failure [40, 41]. Abnormally expressed circRNAs mediate atherosclerotic lesion development via binding with miRNAs or proteins. For example, circWDR77 silencing could alleviate the abnormal proliferation and migration of VSMCs, which was induced by high levels of glucose, via the miR-124/FGF2 axis [42]. circANRIL binds to PES1, stimulates cell apoptosis, and inhibits VSMC and macrophage proliferation [17]. Nevertheless, only a few circRNAs that are involved in atherosclerotic lesions and plaque accumulation have been reported. For the first time, it was verified that circ_0086296 expression is significantly upregulated in human plaque tissues, atherosclerotic mouse aorta, and HUVECs following ox-LDL stimulation. Subsequent functional experiments revealed that circ_0086296 is a promoter for EC injury and atherosclerotic lesion development.

EIF4A3 is the key part of the exon junction complex (EJC), which is responsible for mRNA splicing, transport, and translation. Recent research has found that EIF4A3 facilitates circRNA biogenesis. For example, it was found that EIF4A3 directly combines with the MMP9 mRNA transcript, accelerates circMMP9 cyclization, and increases circMMP9 levels in glioblastoma multiforme [43]. The cyclization of hsa_circ_001988 stimulated by EIF4A3 decreases gastric cancer development by sponging miR-197-3p [44]. In this study, we found that EIF4A3 could directly combine with flanking sites of circ_0086296. Our results also support the finding that EIF4A3 participates in the formation of circ_0086296. Subsequently, EIF4A3 overexpression could enhance circ_0086296 levels, and EIF4A3 knockdown inhibited circ_0086296 levels. These results indicate that EIF4A3 might elevate circ_0086296 levels in HUVECs. Furthermore, we showed that circ_0086296 was colocalized with EIF4A3 in human plaque tissues and HUVECs. However, the more detailed mechanisms of this process require further investigation.

Increasing evidence indicates that circRNA-mediated ceRNA crosstalk plays a vital role in the pathology of cardiovascular diseases [45, 46]. The miRNA sponge role of circ_0086296 was validated in this study. First, we found that circ_0086296 has a target site, miR-576-3p. Second, miR-576-3p level could be regulated by circ_0086296. Third, circ_0086296 and miR-576-3p were colocalized in the cytoplasm of HUVECs. We also found that miR-576-3p inhibitor abolishes the repressive effect of circ_0086296 downregulation on IFIT1 levels. Finally, circ_0086296 facilitates the atherosclerotic ECs phenotype by targeting IFIT1 via miR-576-3p. Thus, our results identified a ceRNA network between circ_0086296, miR-576-3p, and IFIT1 in HUVECs. Notably, a single circRNA can regulate the expression of many downstream genes via sponging multiple miRNAs. Thus, the effect of the circ_0086296-mediated ceRNA sponge network on atherosclerotic lesions still requires further investigation.

High IFIT1 levels have been shown to serve as a useful marker in experimental atherosclerotic animals or clinical pathological applications [47]. Moreover, IFIT1 was involved in LPS-stimulated inflammatory factor expression in HUVECs. The IFIT1 level and proinflammatory cytokine production were also increased in the aortic plaques of pristane-treated ApoE^−/−^ mice [47]. These findings provide more evidence to support that the IFIT1 axis regulates atherosclerotic inflammatory response. We found that circ_0086296 inhibition mitigated proinflammatory cytokine generation in ox-LDL-treated HUVECs by reducing IFIT1 levels. IFIT1 could regulate the STAT1/STING/IRF-3 axis and STING can activate the downstream transcription factors including IRF3 and NF-κB [48–50]. Recent research has revealed that STING knockdown decreases atherosclerotic lesions and inflammatory factor expression in the aorta of atherosclerotic mice. Thus, blockade of STING signals may be a new effective pharmacological target for AS treatment. In this study, we demonstrated that loss of circ_0086296 inhibited ox-LDL stimulated IFIT1/STAT1/STING expression, which was validated both in vitro and in vivo. Despite these findings, more research is needed to identify how the IFIT1/STAT1/STING axis affects atherosclerotic lesions.

Furthermore, the transcription factor STAT1 could promote the transcription of UHRF2. Our data are consistent with the previous finding that transcription factors, such as STAT1 [51], E2F1 [25], and Twist1 [52], may induce the gene transcription via binding to the promoter area. Thus, these transcription factors could increase both gene and circRNA expression. Nevertheless, the molecular mechanisms in circRNA formation require further elucidation. Interestingly, STAT1 could bind to the promoter region of UHRF2, constructing the circ_0086296/miR-576-3p/IFIT1/STAT1 feedback loop in the atherogenic endothelial phenotype progression.

It is broadly known that circRNAs are more stably and specifically expressed in cells. Our results showed that circ_0086296 level is overexpressed in the serum EVs of patients with AS compared with that in the serum EVs of the control donors. Our data also revealed that higher circ_0086296 expression is observed in ox-LDL-treated HUVEC-derived EVs than in untreated cells. To further explore whether circ_0086296 exerts its function via EV transmission, the EVs derived from sh-circ_0086296 or circ_0086296-OE cells were cocultured with HUVECs. We found that sh-circ_0086296 cell-derived EVs decreased the atherosclerotic lesion phenotype of HUVECs. Additionally, circ_0086296-OE cell-derived EVs promoted the atherosclerotic lesion phenotype of HUVECs. Our data suggest the potential role of circ_0086296 in AS development.

## Conclusions

**Fig. 10:**
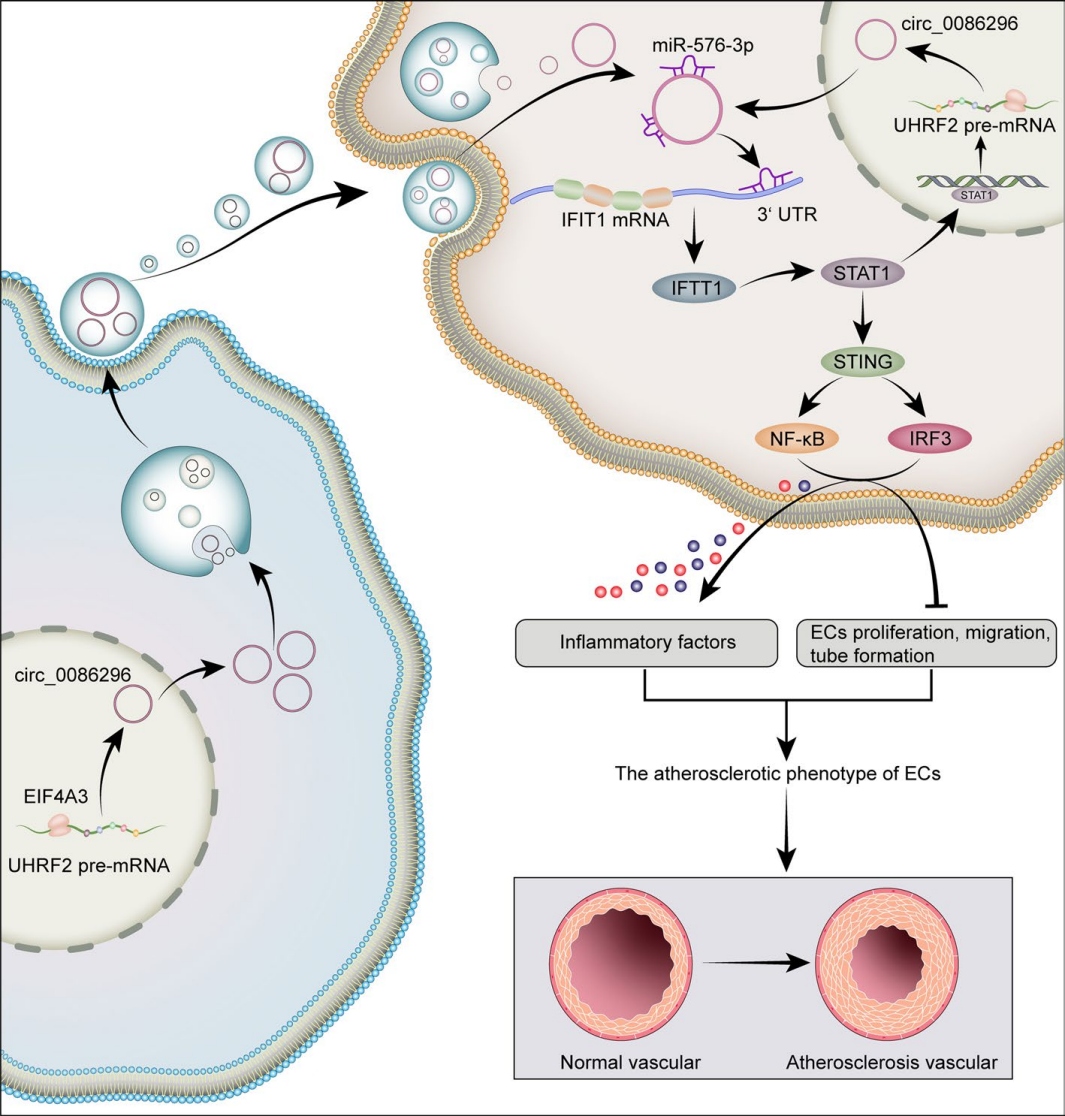
Schematic illustration of circ_0086296-induced atherosclerotic lesions via miR-576-3p/IFIT1/STAT1 feedback loop

In summary, we demonstrated that circ_0086296 is significantly overexpressed in human carotid artery plaques, ox-LDL-treated HUVECs, and the aortas of atherosclerotic mice. Moreover, the EIF4A3-induced circ_0086296 level and circ_0086296/miR-576-3p/IFIT1/STAT1 feedback loop aggravates the atherosclerotic lesion phenotype of HUVECs. Additionally, EVs containing circ_0086296 mediated cell communication and induced the atherosclerotic lesion phenotype of ECs (Fig. 10). To the best of our knowledge, our study is the first to provide evidence of the regulatory mechanism of circ_0086296, thereby providing a theoretical basis for understanding the pathogenesis of AS.

## Supplementary Information

**Additional file 1.** Additional figures S1–S10.

**Additional file 2: Table S1.** Primer sequences for RT-PCR and qPCR analysis.

**Additional file 3.** Basic characteristics of the differently expressed circRNAs.

**Additional file 4.** Basic characteristics of the differently expressed mRNAs.

**Additional file 5.** The binding site of circRNAs and miRNAs.

**Additional file 6.** The binding site of mRNAs and miRNAs.

## Data Availability

The data used and/or analyzed during the current study are available from the corresponding author on reasonable request.

## Abbreviations

AS: Atherosclerosis
ANOVA: Analysis of variance
circRNA: Circular RNA
EC: Endothelial cell
EIF4A3: Eukaryotic initiation factor 4A-III
EJC: Exon junction complex
EV: Extracellular vesicle
FISH: Fluorescent in situ hybridization
HE: Hematoxylin–eosin
HFD: High-fat diet
IF: Immunofluorescence
IFIT1: Interferon-induced protein with tetratricopeptide repeats 1
IHC: Immunohistochemistry
IL: Interleukin
Ox-LDL: Oxidized low-density lipoprotein
NC: Normal control
ND: Normal diet
qRT-PCR: Quantitative real-time polymerase chain reaction
RBP: RNA-binding protein
VSMC: Vascular smooth muscle cells
