# Supplementary material for: circ_0086296 induced atherosclerotic lesions via the IFIT1/STAT1 feedback loop by sponging miR-576-3p
Source: Cell Mol Biol Lett. 2022 Sep 23;27:80. doi: 10.1186/s11658-022-00372-2 (PMC9502643; doi:10.1186/s11658-022-00372-2)
Supplement: Supplementary file 1 — Additional file 1. Additional figures S1–S10. [file 11658_2022_372_MOESM1_ESM.docx]

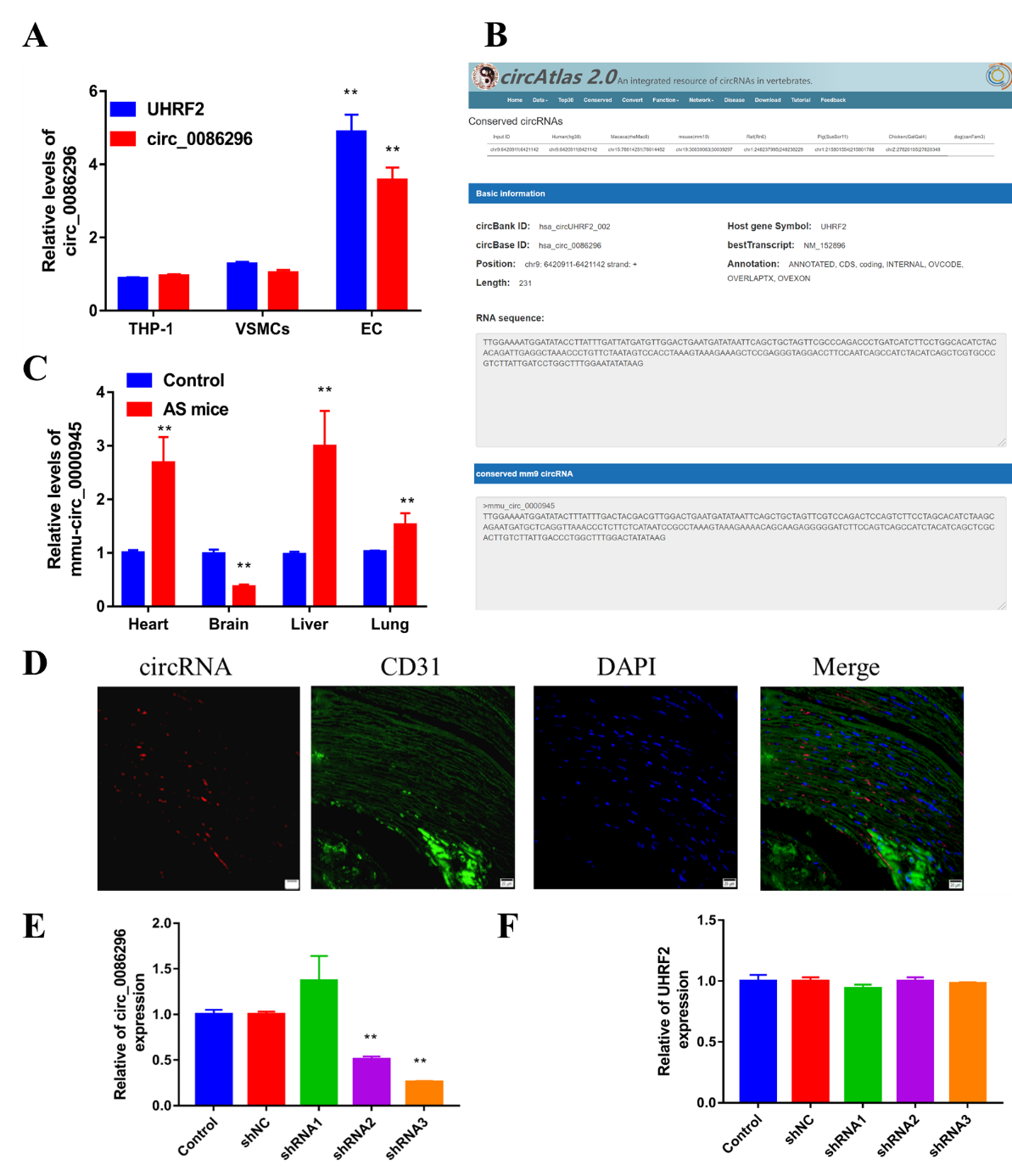


Fig.S1 Validation and characterization of circ_0086296. (A) qRT-PCR validated the circ_0086296 level in different vascular cells. (B) A diagram of hsa_ circ_0086296 and its homologous circRNA mmu-circ_0000945 in the mouse. (C) The circ_0086296 levels was detected in different organ tissues. (D) The colocalization of circ_0086296 with ECs marker in artery tissues has been presented. (E, F) The level of circ_0086296 and UHRF2 were detected after knockdown of circ_0086296. **p < 0.001


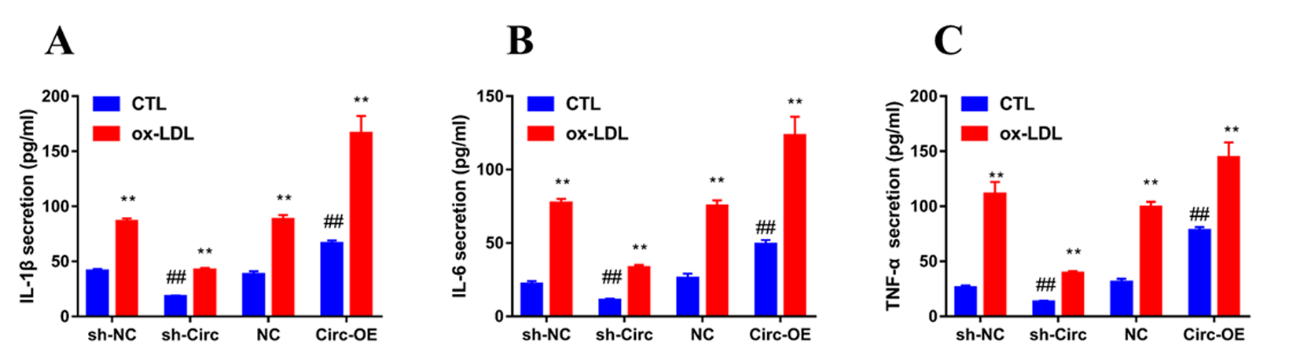


Fig.S2 The IL-1β (A), IL-6 (B) and TNF-α (C) levels in HUVECs infected with circ_0086296 overexpression vector or sh-circ_0086296 vectors was measured via ELISA. **p < 0.001, ^##^p < 0.001


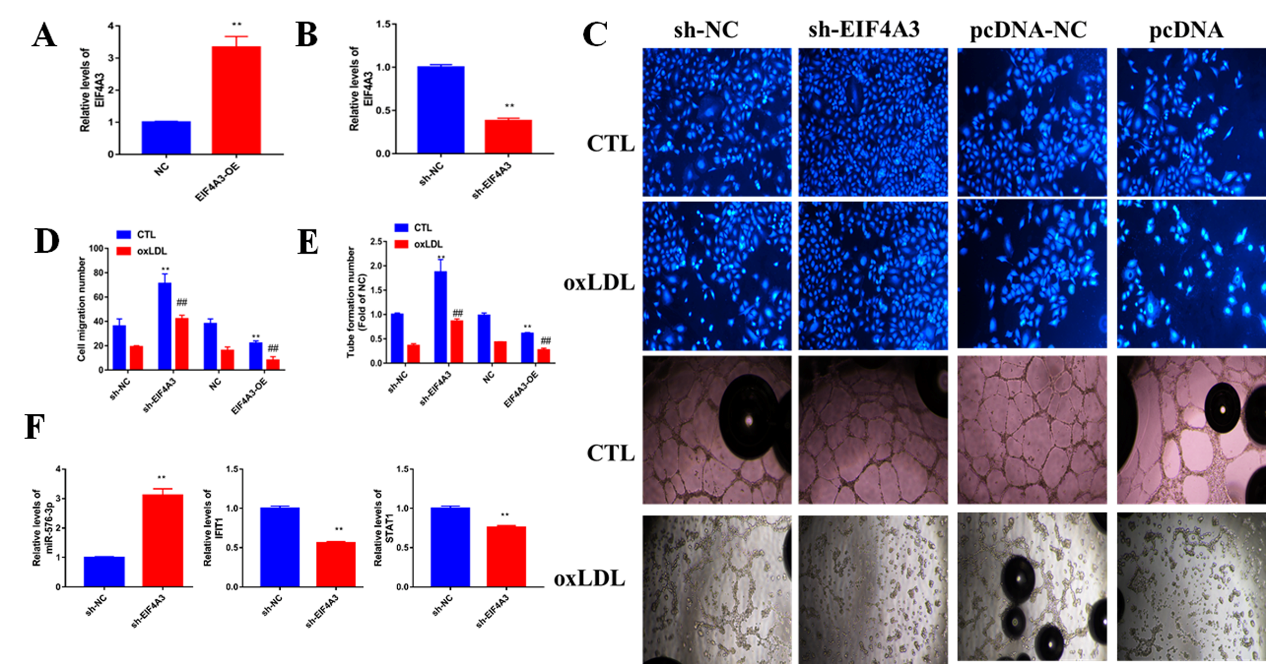


Fig.S3 The effects of EIF4A3 on the oxidized low-density lipoprotein (ox-LDL)-induced atherosclerotic phenotype in HUVECs. (A, B) The effects of EIF4A3 overexpression or sh-EIF4A3 were measured via qRT-PCR. (C, D) The migration of HUVECs transfected with EIF4A3 overexpression or sh-EIF4A3 vectors was determined via Transwell assay. (C, E) The angiogenic ability of HUVECs infected with EIF4A3 overexpression or sh-EIF4A3 vectors was measured via Matrigel assay. (F) The effects of sh-EIF4A3 on miR-576-3p/IFIT1/STAT1 was presented. **p < 0.001, ^##^p < 0.001


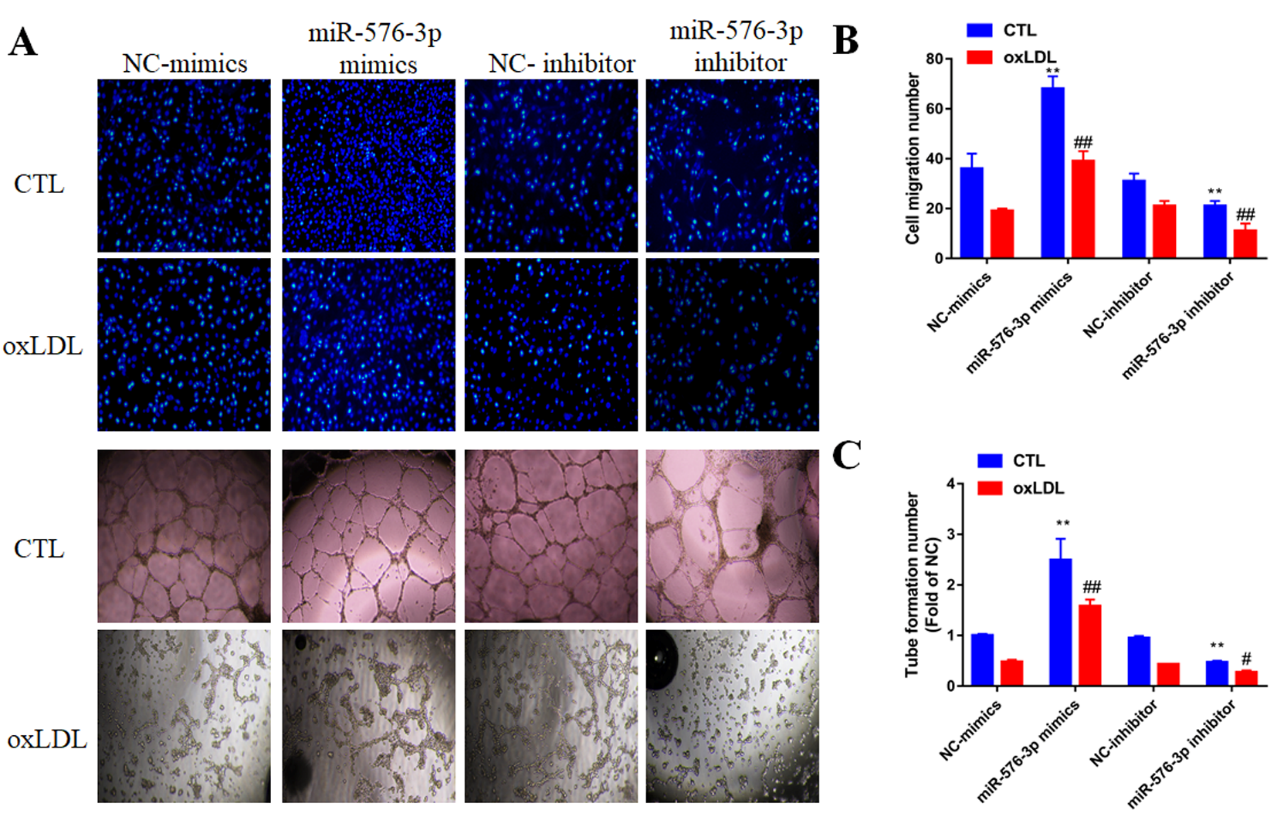


Fig.S4 The effects of miR-576-3p on the ox-LDL-induced atherosclerotic phenotype in HUVECs. (A, B) The migration of HUVECs transfected with miR-576-3p mimics or inhibitor was determined via Transwell assay. (A, C) The angiogenic ability of HUVECs infected with miR-576-3p mimics or inhibitor was measured via Matrigel assay. **p < 0.001, ^##^p < 0.001


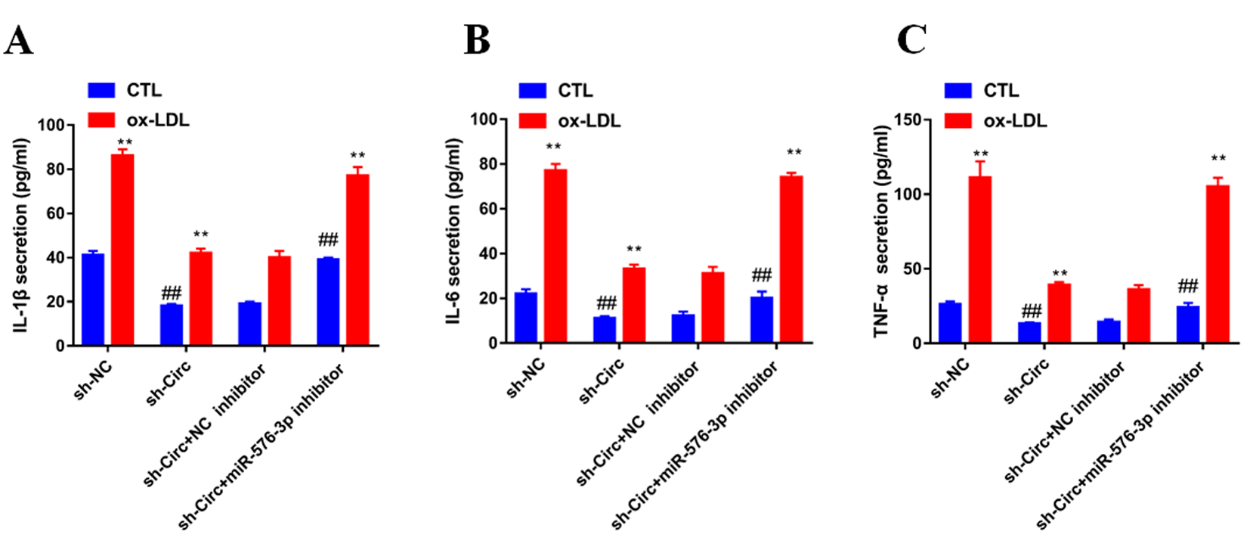


Fig.S5 The IL-1β (A), IL-6 (B) and TNF-α (C) levels in HUVECs infected with sh-circ_0086296 vectors and miR-576-3p inhibitor was measured via ELISA. **p < 0.001, ^##^p < 0.001


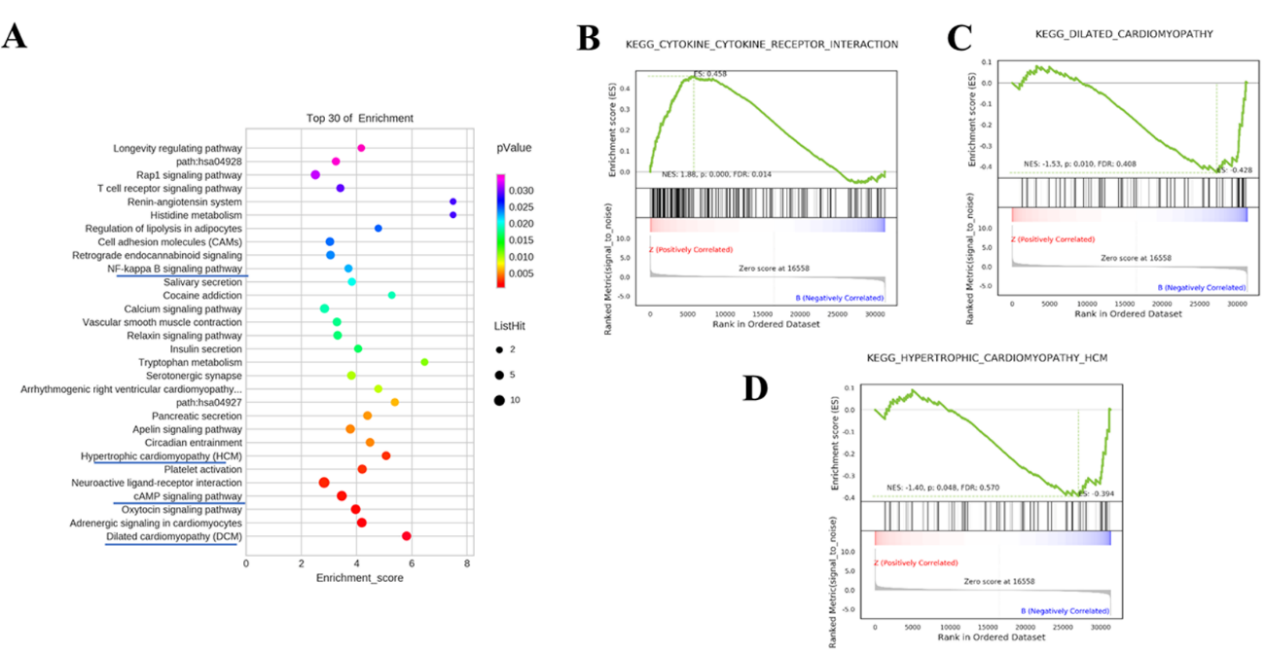


Fig. S6 KEGG enrichment and Gene set enrichment analysis of pathways in the human plaque tissues and control samples.


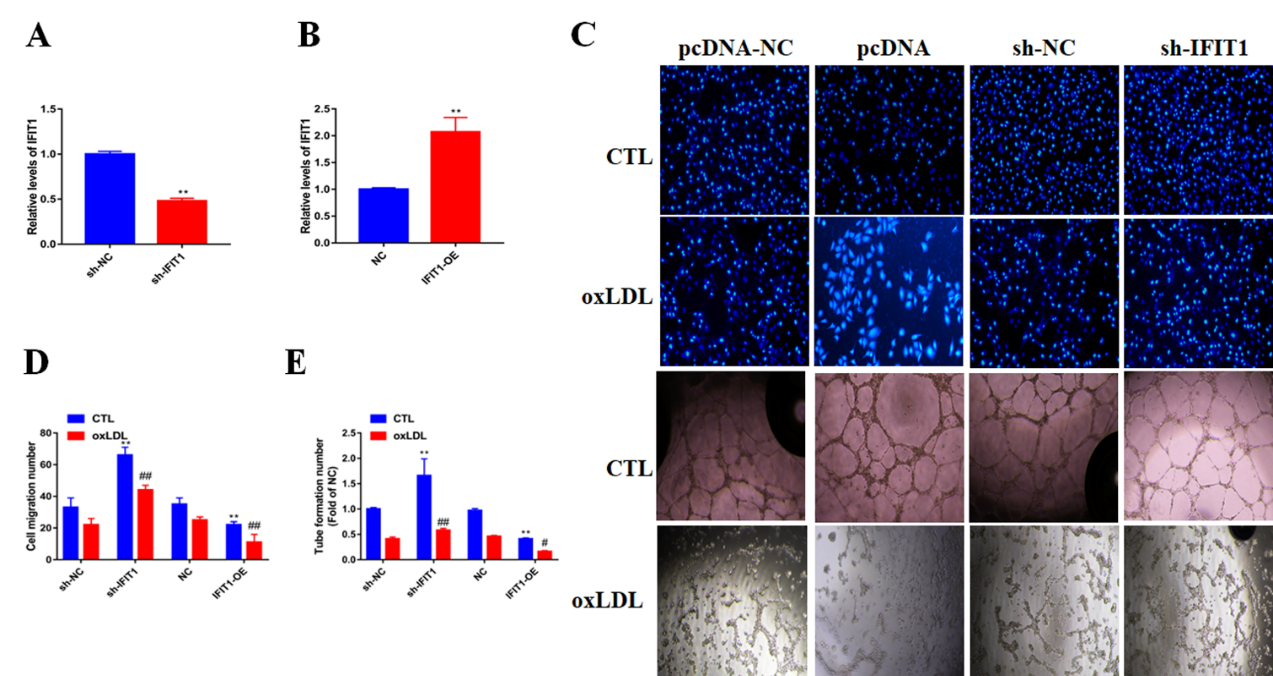


Fig.S7 The effects of IFIT1 on the ox-LDL-induced atherosclerotic phenotype in HUVECs. (A, B) The effects of IFIT1 overexpression or sh-IFIT1 were measured via qRT-PCR. (C, D) The migration of HUVECs transfected with IFIT1 overexpression vector or sh-IFIT1 vectors was determined via Transwell assay. (C, E) The angiogenic ability of HUVECs infected with IFIT1 overexpression vector or sh-IFIT1 vectors was measured via Matrigel assay. **p < 0.001, ^##^p < 0.001


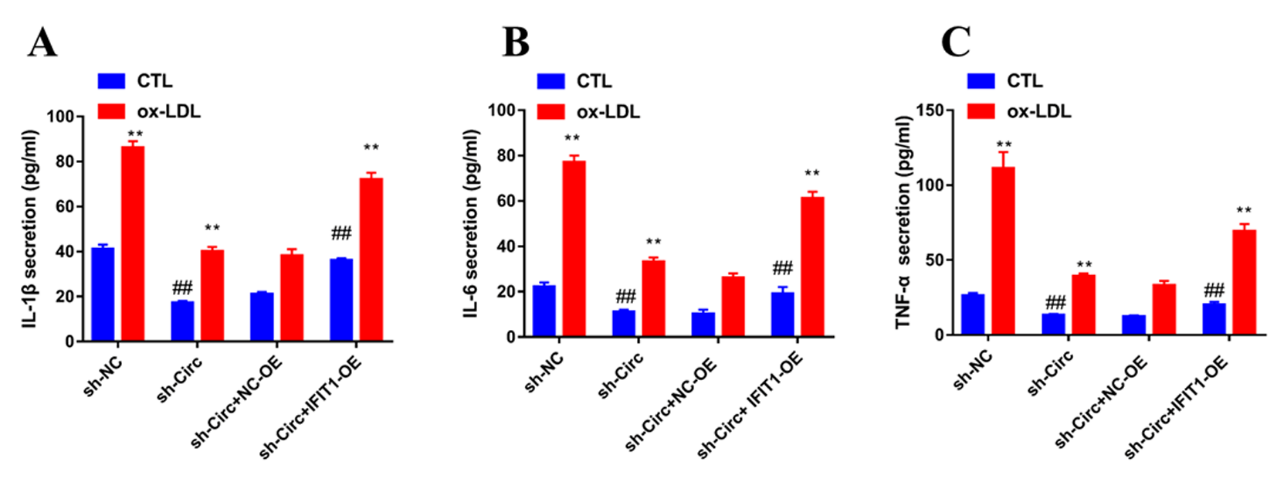


Fig.S8 The IL-1β (A), IL-6 (B) and TNF-α (C) levels in HUVECs infected with sh-circ_0086296 vectors and IFIT1 overexpression vector was measured via ELISA. **p < 0.001, ^##^p < 0.001


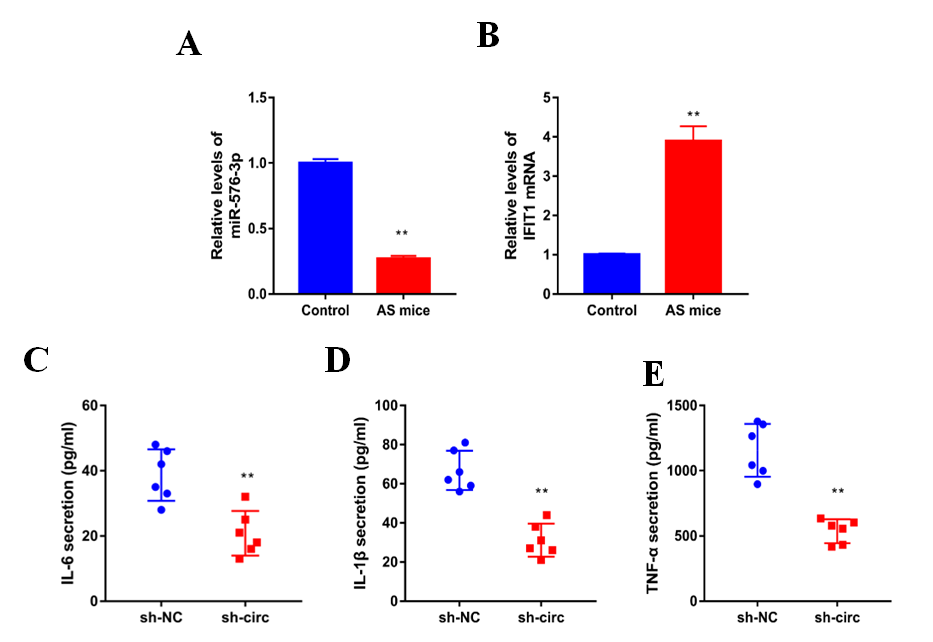


Fig.S9 Circ_0086296 promoted the atherosclerotic phenotype *in vivo*. The expression levels of miR-576-3p (A) and IFIT1 (B) in AS mice were determined by qPCR. The IL-6 (C), IL-1β (D) and TNF-α (E) levels in AS mice infected with sh-circ_0086296 vectors was measured via ELISA. **p < 0.001


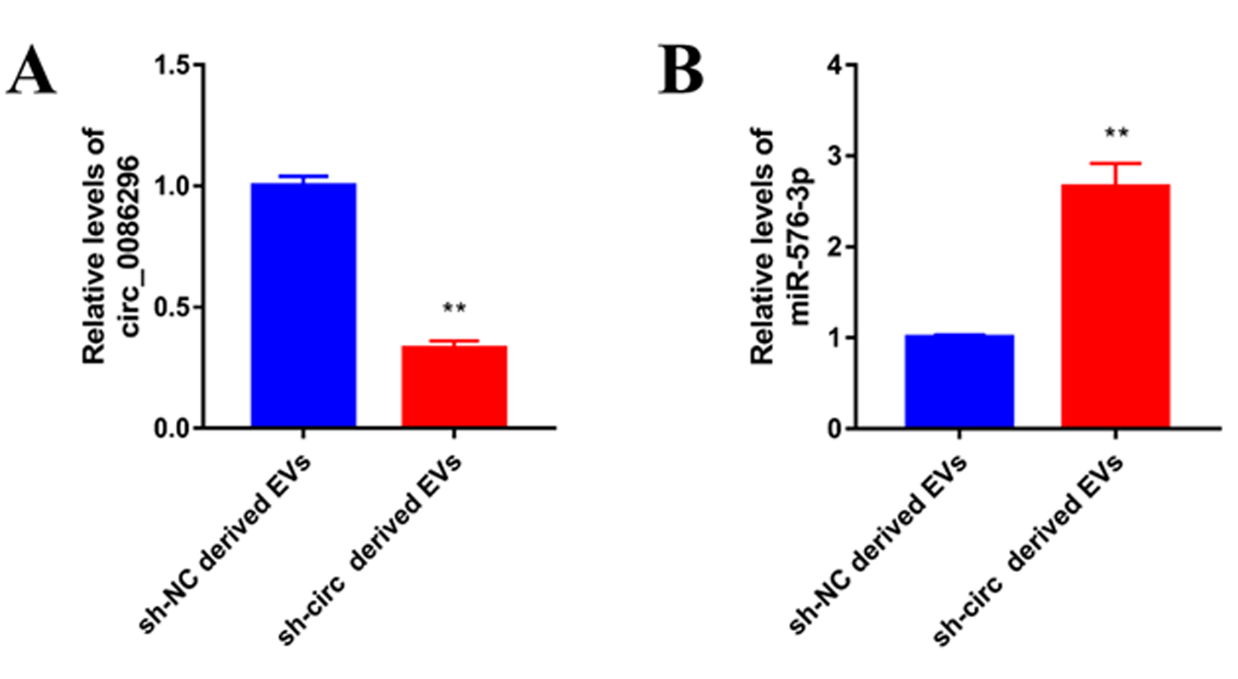


Fig.S10 QRT-PCR analysis of circ_0086296 (A) and miR-576-3p (B) expression in the cells treated with EVs originating from HUVECs infected with sh-circ_0086296 vectors. **p < 0.001
