# Supplementary material for: circ_0086296 induced atherosclerotic lesions via the IFIT1/STAT1 feedback loop by sponging miR-576-3p
Source: Cell Mol Biol Lett. 2022 Sep 23;27:80. doi: 10.1186/s11658-022-00372-2 (PMC9502643; doi:10.1186/s11658-022-00372-2)
Supplement: Supplementary file 2 — Additional file 2: Table S1. Primer sequences for RT-PCR and qPCR analysis. [file 11658_2022_372_MOESM2_ESM.docx]

| **Primer name** | **Forward (5’ - 3’)** | **Reverse (5’ - 3’)** |
| --- | --- | --- |
| circ_0086296 | CGTGCCCGTCTTATTGATCC | CTGGGCGAACTAGCAGCTGA |
| UHRF2 | TTCTTGCTCCTGTCGTGTATGT | CTTGAGTCTTTCACCAGCCTTT |
| GAPDH | GTCTCCTCTGACTTCAACAGCG | ACCACCCTGTTGCTGTAGCCAA |
| miR-576-3P | AAGATGTGGAAAAATTGGAATC |  |
| IFIT1 | AGAAGCAGGCAATCACAGAAAA | CTGAAACCGACCATAGTGGAAAT |
| EIF4A3 | GGGGCATCTACGCTTACGG | GCGATGACATCTCTCCCTTTGA |
| **microRNA sequence**  mimics NC ACUACUGAGUGACAGUAGA  miR-576-3p mimics AAGAUGUGGAAAAAUUGGAAUC  inhibitor NC CAGUACUUUUGUGUAGUACAA  miR-576-3p inhibitor GAUUCCAAUUUUUCCACAUCUU | |  |

**Table S1. Primer sequences for RT-PCR and qPCR analysis.**
